# Understanding Selectivity in Product Distributions from Laser Ablation of Organic Liquids

**DOI:** 10.1021/acs.jpcb.4c05638

**Published:** 2024-10-16

**Authors:** Samuel Harris, Ella Kaplan, Michael Aftel, Katharine Moore Tibbetts

**Affiliations:** Department of Chemistry, Virginia Commonwealth University, Richmond, Virginia 23284, United States

## Abstract

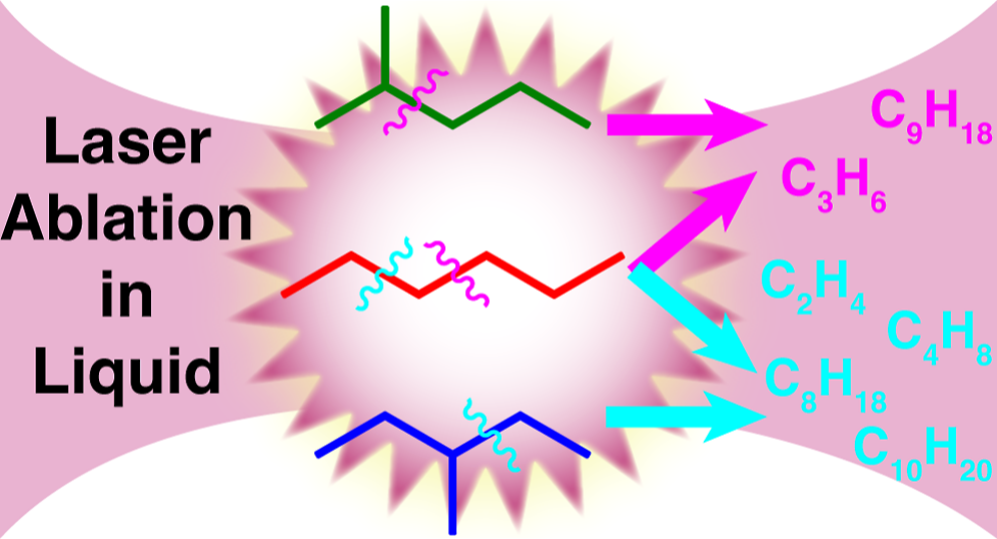

Pulsed laser ablation in organic solvents is widely used to produce oxide-free metal and metal carbide nanoparticles, often with carbon coatings resulting from laser-induced reactions in the organic solvent. To gain insight into how the molecular structure of the solvent affects these reaction pathways, this work investigates ablation of the C_6_H_14_ isomers *n*-hexane, 2-methylpentane, and 3-methylpentane through characterization of the gas and liquid products with mass spectrometry. Ablation of each C_6_H_14_ isomer produces a distinct distribution of product molecular weights and isomers. 2-methylpentane preferentially produces C_3_ and C_9_, whereas 3-methylpentane produces C_2_, C_4_, C_8_, and C_10_ products. These preferential product distributions, along with the lack of such selectivity in *n*-hexane, arise from differences in the most favorable C–C bond scission pathways in each C_6_H_14_ isomer. Moreover, the particular isomers of C_8_H_18_, C_9_H_20_, C_10_H_22_, and C_12_H_26_ produced by ablation of each C_6_H_14_ isomer indicate that the vast majority of reaction pathways involve addition reactions between a fragment radical and parent C_6_H_14_ or between two C_6_H_14_ molecules, without molecular rearrangement. This propensity toward direct addition suggests that the chemical reactions induced by ultrashort pulsed laser ablation proceed on faster time scales than those of radical rearrangements.

## 1 Introduction

Pulsed laser excitation of liquid media is widely used to synthesize colloidal metal and semiconductor nanoparticles^1−6^ and in recent years has emerged as a “green” technique for catalyst-free H_2_ production from water^7^ and aqueous ammonia.^8^ Although typical laser synthesis and processing of colloids (LSPC) methods involve ablation of a bulk or powdered metal target in water or aqueous solution, there has been increasing interest in using organic liquids for LSPC to limit metal oxidation, produce metal carbide phases, and generate protective carbon shells around the nanoparticles.^9−17^ The use of organic liquids for nanoparticle production by LSPC is widely known to generate byproduct gases including H_2_, CO, and small hydrocarbons,^18−24^ and carbon nanoparticle byproducts have also been reported.^25,26^ Ablation of neat organic liquids also produces gas, liquid, and solid products.^27−34^ Despite the numerous investigations of how organic liquids influence the properties of nanoparticles generated by LSPC and growing attention to characterization of molecular byproducts, it remains challenging to elucidate the reaction mechanisms driving byproduct formation due to the complexity of laser-induced chemical reactions in organic liquids.^35^

The physicochemical processes induced by laser–material interaction in water involve the formation of plasma containing electrons and radicals (OH^•^ and H^•^).^5,36^ In contrast to the few reactive species produced in water, the laser-induced decomposition of organic liquid molecules produces many transient reactive species including C_2_,^37,38^ CH,^37,39^ and solvent molecule radicals and cations.^39,40^ Chemical reactions in LSPC involving these and other reactive species have been proposed to take place in the gas phase within the cavitation bubbles formed immediately following laser excitation.^16−18^ As a result, the formation of molecular byproducts during LSPC in organic liquids is often rationalized in terms of free-radical gas-phase pyrolysis reactions.^22,23,35,41,42^ However, recent studies of femtosecond and few-picosecond laser ablation of neat organic solvents have reported product distributions that are inconsistent with the nonselective radical chemistry observed under pyrolysis conditions. Instead, the major liquid products are molecular dimers formed by direct bond formation between solvent molecules, without the molecular rearrangement that would be expected from long-lived free radicals.^31−34^ Ishikawa and Sato have attributed these solvent-specific reactivity patterns to mechanically induced bond-formation between two solvent molecules by laser-driven shock waves.^31−33^ Alternatively, the observed reactivity patterns could arise from reactions in the laser plasma involving radicals and ions because the specific molecular dimer isomers that were recently observed in ultrashort pulsed laser ablation of *n*-hexane^31,34^ match those observed decades ago in γ radiolysis studies.^43−46^ Similar product distributions as in γ radiolysis have also been observed in femtosecond laser ablation of isopropyl alcohol/water mixtures.^24^

To assess contributions from the competing pictures of gas-phase pyrolysis, shock wave-driven bond formation, and ion/radical chemistry to the product distributions from pulsed laser ablation, this work compares the ablation products of three hexane (C_6_H_14_) isomers: *n*-hexane, 2-methylpentane, and 3-methylpentane. Comparison of ablation products from isomeric species provides a unique opportunity to extract information about the specific structures of transient carbon radicals present in the laser plasma and their reactions with parent solvent molecules. Moreover, hexanes constitute a common liquid for LSPC applications,^10,11,23^ although they are used less often than solvents with lower carbon and oxygen content like acetone and ethanol.^4^ Finally, as components of hydrocarbon fuels, hexane isomers have been subject to radiolysis,^43−48^ pyrolysis,^49,50^ and plasma discharge^51,52^ studies, enabling direct comparison of product distributions between these methods and pulsed laser ablation.

To specifically assess the relative contributions of plasma reactions and laser-induced shock waves, we conduct ablation with both typical tightly focused conditions^31−34^ and a collimated femtosecond laser beam that produces low-density plasma (LDP).^36,53^ By operating below the electron density threshold for cavitation bubble formation (1.8 × 10^20^ cm^–3^), LDP avoids thermo-mechanical effects including shock wave and cavitation bubble generation.^36,53^ Our observation of similar product distributions upon ablation with LDP and focused pulses with the same 30 fs duration indicates that chemical reactions of ions and radicals in the laser plasma, rather than shock waves, primarily determine the product distributions under femtosecond excitation. In contrast, changes in product distributions upon ablation with focused picosecond pulses point to a greater role for shock waves under these conditions.

Understanding how the structure of liquid organic molecules impacts the product distributions from pulsed laser ablation is important for advancing nanoparticle synthesis by LSPC. For instance, the molecular structure of the liquid impacts LSPC metal nanoparticle productivity according to a recent study that observed twice the rate of gold nanoparticle production and half the yield of gas byproducts from ablation of a gold target in *n*-hexane compared to ablation in isohexane (92% methylpentane isomers).^23^ In this work, the observation of isomer-specific product distributions that correlate with observed gas-phase dissociation pathways upon strong-field ionization (SFI)^54,55^ in each hexane isomer suggests that the susceptibility to cleavage of different C–C bonds in a given molecular structure directly determines laser ablation product distributions. These findings can aid in future choice of LSPC liquid media to tailor nanoparticle properties.

## 2 Experimental Methods

### 2.1 Materials

*n*-hexane (99%, Millipore Sigma), 2-methylpentane (98%, TCI), 3-methylpentane (99%, Thermo Scientific), acetophenone (99%, Sigma-Aldrich), PIANO isoparaffins mixture 90 (2-H492031NA, Dr. Ehrenstorfer) were used as received without further purification.

### 2.2 Laser Ablation in Liquid

The experimental setups have been described in detail previously.^34,56,57^ Briefly, a commercial titanium-sapphire chirped-pulse amplifier (Astrella, Coherent, Inc., Santa Clara, CA, USA) producing 30 fs, 800 nm pulses at a repetition rate of 1 kHz was used. The pulse width was controlled by detuning the compressor grating from its zero-dispersion position (30 fs pulse width) to produce negatively chirped 4 ps pulses. Laser ablation was carried out under two conditions: (1) 1 mJ pulses tightly focused with a *f* = 5 cm aspheric lens, using either 30 fs or 4 ps pulses;^34,56^ or (2) 2.6 mJ, 30 fs pulses, collimated to a beam waist of 5.8 mm, to produce LDP through supercontinuum generation in the liquid sample.^57^ The beam waist of the tightly focused pulses was previously measured to be 6.52 μm,^56^ producing calculated focal intensities (excluding losses) of 2.5 × 10^16^ W cm^–2^ at 30 fs and 1.9 × 10^14^ W cm^–2^ at 4 ps. It should be noted that the actual peak intensity is likely much lower for focused 30 fs pulses in particular due to losses from supercontinuum generation and filamentation.^58^ The LDP condition produces estimated peak intensity of approximately 10^13^ W cm^–2^ upon self-focusing and filamentation.^53,57^ The high pulse energy used results in immediate filamentation at the cuvette–liquid interface as observed previously,^58^ and the filaments extend through the entire 1 cm cuvette path length. For each laser condition, liquid samples were ablated for 30 min under vacuum (see Section 2.3 below) to avoid production of oxidized products that were observed in previous work.^31,34^

### 2.3 Mass Spectrometry

2.3.1

SFI-mass spectrometry (SFI-MS) of headspace gas was conducted using 1800 nm, 20 fs pulses for ionization using a similar setup as described previously.^55^ Briefly, a portion of the titanium-sapphire output at 800 nm was converted to 1800 nm by taking the idler output from an optical parametric amplifier. The resulting beam was focused into the extraction region of a linear time-of-flight mass spectrometer (Jordan TOF, Grass Valley, CA). A vacuum-compatible quartz cuvette containing 3 mL of target liquid was attached to an ultrahigh vacuum chamber (base pressure 3 × 10^–9^ Torr) and evacuated through a variable effusive leak valve for 30 min to remove air from the sample. Air removal was confirmed by the low N_2_ and O_2_ signals that were of similar magnitude to the background signals obtained when evacuating an empty cuvette. After evacuation, the pressure of the remaining headspace gas from the liquid sample was set to 8 × 10^–8^ Torr for analysis. Mass spectra were recorded by averaging over 50,000 laser shots using a 1 GHz digital oscilloscope (WaveRunner 610Zi, Teledyne LeCroy). Spectra were normalized to the area of the C_6_H_14_ parent ion signal at *m*/*z* 86. SFI mass spectra were recorded for each sample before laser ablation and then again after ablation for 30 min to measure the formation of new gaseous products.

2.3.2

For liquid product analysis, ablated samples were passed through a 0.22 μm filter (Restek) with the aid of a 5 mL syringe (BD Luer-Lok) to remove any solids. Gas chromatography mass spectrometry (GC–MS) was performed on an Agilent 7890A GC System with a HP 5973 mass selective detector equipped with an HP-5MS UI column. Data were collected with MSD ChemStation F.01.01.2317. The GC oven was set to ramp the temperature from 40 to 130 °C at a rate of 5 °C per minute.

## 3 Results

Laser ablation of each hexane liquid was carried out under three conditions: LDP, focused 30 fs pulses, and focused 4 ps pulses. Because LDP induces plasma chemistry without concomitant thermo-mechanical effects (i.e., shock waves and cavitation bubbles), it provides a baseline to assess the relative contributions of plasma and shock waves to the product distributions. Mass spectra and GC chromatograms for LDP are reported in the main text and the data for tightly focused pulses are provided in the Supporting Information. This section is organized as follows: Section 3.1 describes the gas-phase decomposition patterns of each hexane isomer when subject to SFI, Section 3.2 reports the gaseous products generated by laser ablation in liquid using SFI-MS measurements, and Section 3.3 reports the liquid products of laser ablation obtained from GC–MS measurements.

### 3.1 Decomposition of Gas-Phase Hexane Isomers

**Figure 1:**
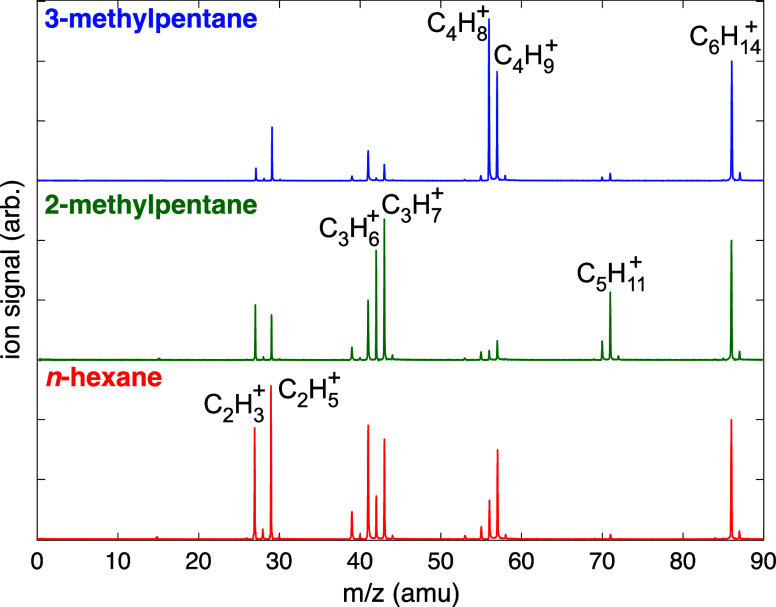
SFI mass spectra of *n*-hexane (red), 2-methylpentane (green), and 3-methylpentane (blue) recorded with 1800 nm, 7 × 10^13^ W cm^–2^, 25 fs pulses. Formulas of selected peaks are labeled.

As a baseline comparison to laser ablation in the liquid hexanes, SFI-MS^54,55^ was performed to determine the preferred gas-phase decomposition pathways of each isomer when subject to intense pulsed laser excitation. Figure 1 shows the SFI mass spectra of *n*-hexane (red), 2-methylpentane (green), and 3-methylpentane (blue) measured at a moderate laser intensity of 7 × 10^13^ W cm^–2^ that does not create multiply charged ions. Each spectrum shows a significantly higher yield of the intact parent molecular ion (*m*/*z* 86) than observed in electron-impact mass spectra (EI-MS)^59^ because SFI with near-infrared excitation induces “soft” ionization.^54,55^ Otherwise, the strikingly different fragmentation patterns of each isomer resemble those observed in EI-MS, albeit with greater selectivity toward favored fragments. SFI of *n*-hexane produces similar yields of C_2_H_*x*_^+^ (*x* = 3, 5), C_3_H_*x*_^+^ (*x* = 5, 7), and C_4_H_9_^+^. 2-methylpentane produces primarily C_5_H_11_^+^ and C_3_H_*x*_^+^ (*x* = 5–7), with a very low yield of C_4_ fragments. 3-methylpentane, on the other hand, fragments largely into C_4_H_8_^+^ and C_4_H_9_^+^, with low yields of C_5_ and C_3_ fragments.

**Figure 2:**
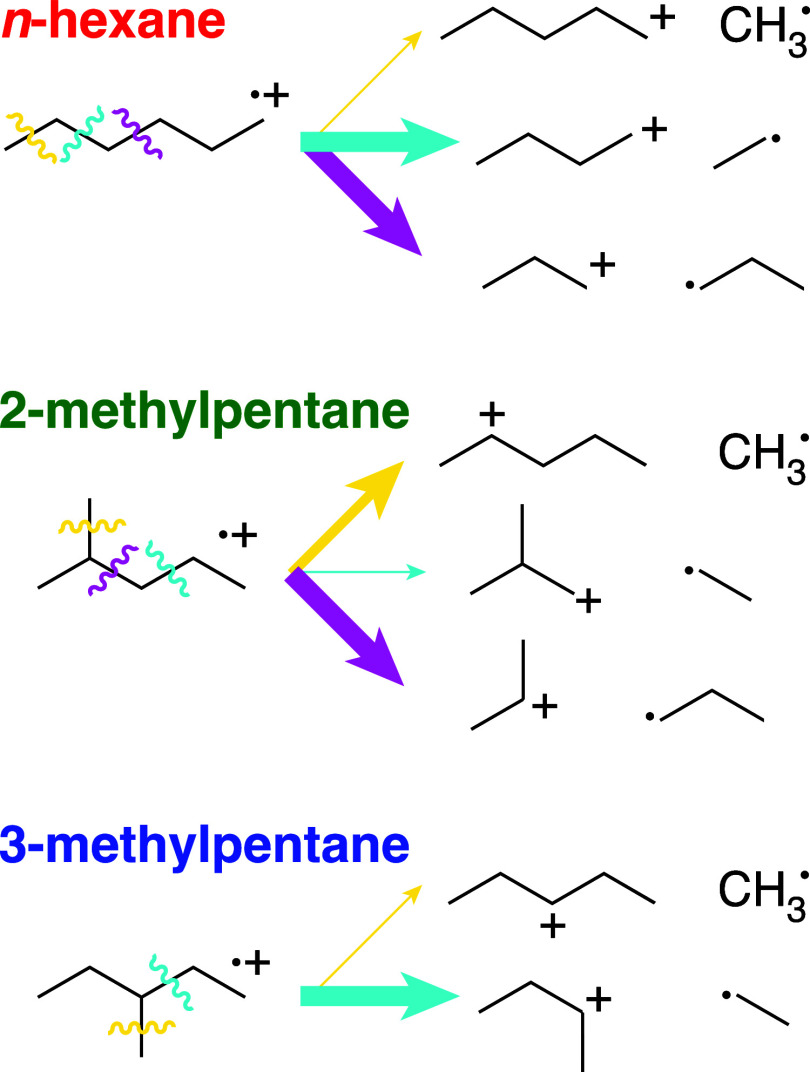
Schematic diagram of dissociation pathways in hexane isomer cations. Colors of squiggly lines and arrows denote cleavage of different bonds to produce C_5_H_11_^+^ + CH_3_^•^ (yellow), C_4_H_9_^+^ + C_2_H_5_^•^ (cyan), and C_3_H_7_^+^ + C_3_H_7_^•^ (magenta). Arrow thickness denotes relative contribution of each dissociation pathway. In 2- and 3-methylpentane, the only yellow pathway shown is that which produces a secondary C_5_H_11_^+^ cation.

The distinct fragmentation patterns of each isomer can be rationalized by considering the substitution patterns of cationic fragments produced upon different C–C bond scission pathways, as shown in Figure 2. In *n*-hexane, all pathways produce primary cations, leading to similar yields of C_3_H_5_^+^, C_3_H_7_^+^, and C_4_H_9_^+^. The lack of C_5_ fragments is likely due to formation of CH_3_^•^ being unfavorable. In contrast, C_5_H_11_^+^ formation in 2-methylpentane is more favorable because CH_3_^•^ loss produces a secondary cation. Formation of a secondary C_3_H_7_^+^ ion also explains the high yield of C_3_H_*x*_^+^ fragments from 2-methylpentane. In 3-methylpentane, dissociation to form C_4_H_9_^+^ also produces a secondary cation, explaining the high yields of C_4_H_9_^+^ and C_4_H_8_^+^. The lack of C_5_H_11_^+^ despite the production of a secondary cation could be due to sequential fragmentation of this cation to form the C_3_ fragments observed in the mass spectrum (no single bond scission can form C_3_ fragments, as evident in Figure 2). Similarly, the C_2_ fragments in all isomers likely arise from sequential fragmentation of larger cationic fragments. In the next section, we will show that the gas product yields from ablation of the liquid hexane molecules result in similar selectivity among C_2_, C_3_, and C_4_ products as observed in these unimolecular dissociation reactions.

### 3.2 Gas Products from Ablation of Hexane Isomer Liquids

**Figure 3:**
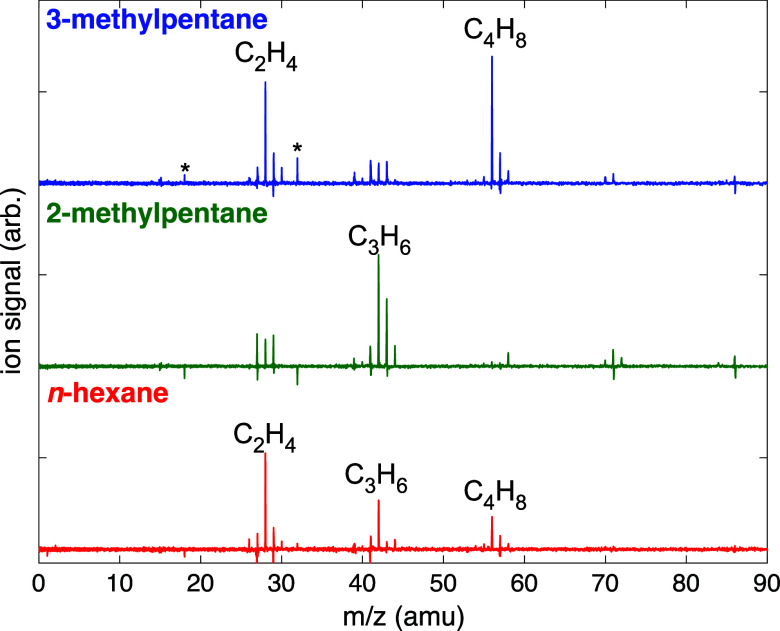
Difference mass spectra for *n*-hexane (red), 2-methylpentane (green), and 3-methylpentane (blue) obtained by SFI with 1800 nm, 1.8 × 10^14^ W cm^–2^ pulses. Major product ions are indicated. The * denotes contaminants from air, H_2_O and O_2_.

SFI-MS were recorded for each liquid prior to ablation and then again after ablation. Difference mass spectra obtained by subtracting the spectrum taken before ablation from the spectrum taken after ablation were used to identify and quantify relative yields of gas products, as described previously.^24,34^Figure 3 shows the difference mass spectra obtained from LDP for each hexane isomer. The raw mass spectra, along with difference mass spectra for ablation with focused pulses, are given in the Supporting Information, Figures S1–S4. From the spectra in Figure 3, it is evident that similar patterns of gaseous product generation emerge as the fragmentation patterns observed with SFI-MS in Figure 1. Compared to the production of C_2_H_4_, C_3_H_6_, and C_4_H_8_ in *n*-hexane, 2-methylpentane is strongly selective toward C_3_H_6_ while 3-methylpentane produces mostly C_2_H_4_ and C_4_H_8_. These patterns are rationalized by the C–C bond cleavage pathways illustrated for hexane cations in Figure 2, where the pathways that produce fragments with a higher degree of substitution are favored.

**Figure 4:**
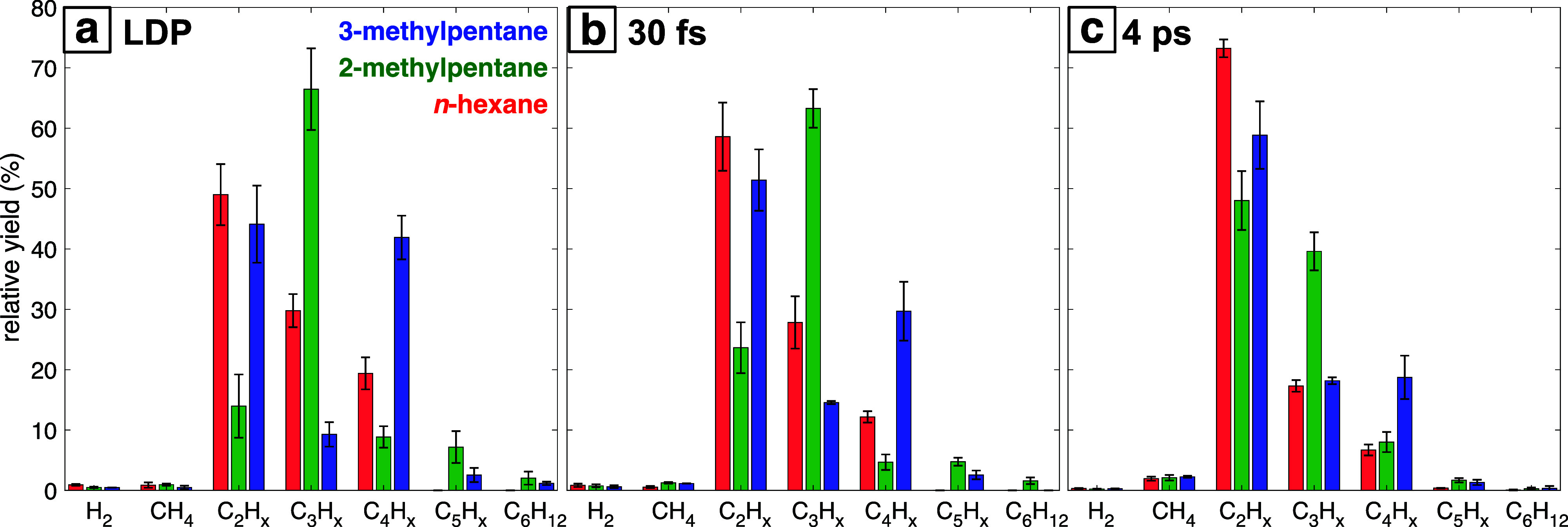
Distribution of gas products obtained after laser ablation with (a) LDP, (b) focused 30 fs pulses, and (c) focused 4 ps pulses. Error bars denote standard deviation over duplicate measurements.

To quantify the relative selectivity toward different gaseous products under each of the three laser conditions (LDP, focused 30 fs, focused 4 ps), the peak areas under each product in the difference mass spectra were integrated and normalized to the total signal from product gases. The resulting distributions of gaseous products are compiled in Figure 4 (tabulated yields are given in the Supporting Information, Tables S1–S3). It should be noted that the relative yields of H_2_ and CH_4_ are very low because their ionization energies (15.43 and 12.61 eV respectively) are much higher than for larger molecules (∼10–11 eV for C_2_ through C_4_ molecules).^59^ Although higher yields of H_2_ and CH_4_ could be obtained by further increasing the laser intensity beyond the high intensity of 1.8 × 10^14^ W cm^–2^ used, this resulted in saturation of the larger fragment ion signals. Additionally, the C_5_ and C_6_ species are mostly liquid at room temperature and rely on vapor pressure to be detected by SFI-MS. Due to these limitations, quantitative estimations of the relative yields of gas products with different ionization energies or volatile liquids with different vapor pressures are not possible. Nevertheless, the similar ionization energies of C_2_ through C_4_ gases allow for qualitative comparison of their relative yields from each hexane isomer. It is evident from Figure 4 that the LDP condition results in strong selectivity toward C_3_ gases in 2-methylpentane compared to C_2_ and C_4_ gases in 3-methylpentane. The product distribution for focused 30 fs pulses is only slightly shifted toward C_2_ gases in all isomers. In contrast, ablation with 4 ps pulses resulted in a strong selectivity toward C_2_ gases (both acetylene and ethylene). This result is consistent with our previous work comparing gas yields from various organic liquids, where picosecond pulses consistently produced higher relative yields of C_2_ gases independent of the initial liquid molecule.^34^ The higher yields of C_2_ gases with picosecond pulses were attributed in the latter work to the higher kinetic energy of plasma electrons and cavitation bubble collapse observed under picosecond ablation,^60,61^ which is expected to enhance molecular decomposition into small fragments that can form C_2_ gases.

### 3.3 Liquid Products from Ablation of Hexane Isomer Liquids

**Figure 5:**
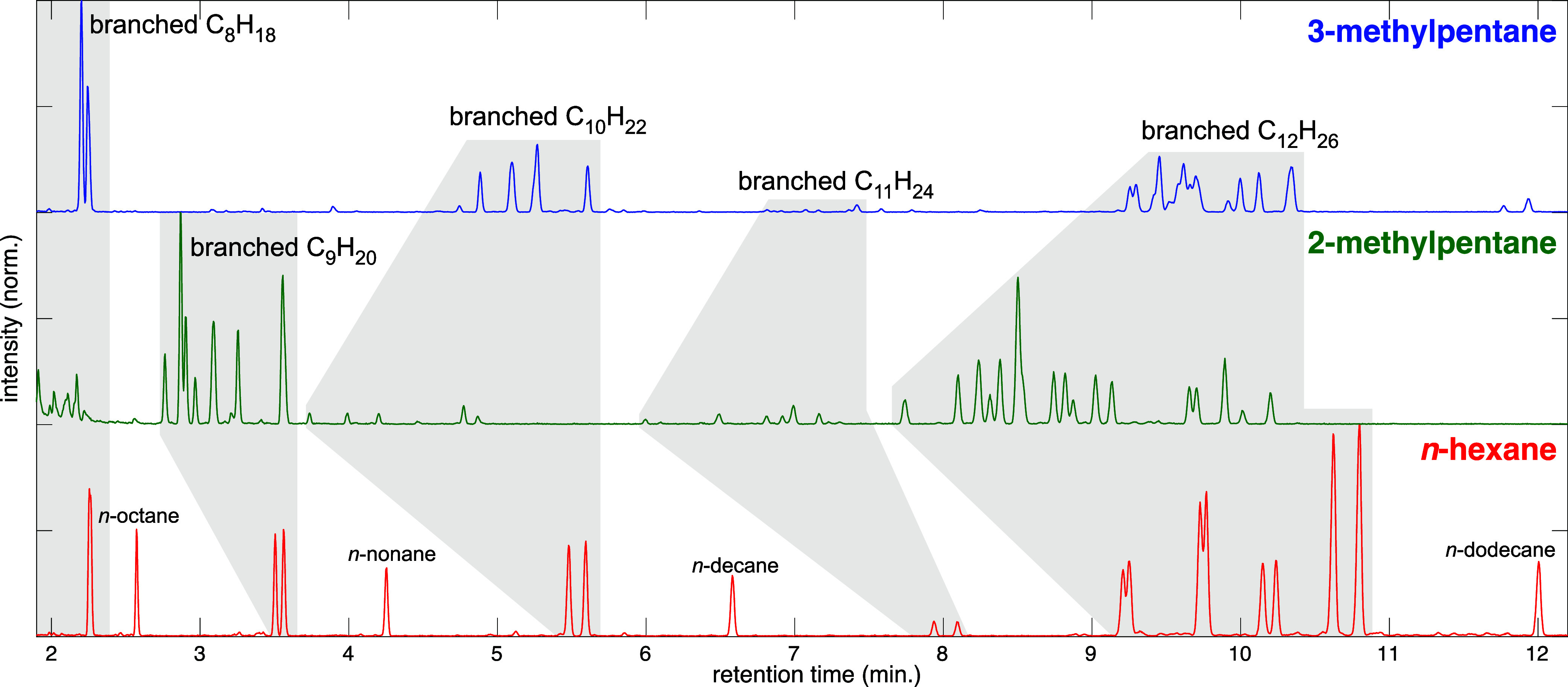
Total ion chromatograms of *n*-hexane (red), 2-methylpentane (green), and 3-methylpentane (blue) ablated with 30 fs, 2.6 mJ pulses under LDP conditions for 30 min. Shaded regions indicate branched C_8_H_18_, C_9_H_20_, C_10_H_22_, C_11_H_24_, and C_12_H_26_ products.

Figure 5 shows the total ion chromatograms obtained from ablation of *n*-hexane (red), 2-methylpentane (green), and 3-methylpentane (blue) under LDP. The chromatograms obtained after ablation with focused 30 fs and 4 ps pulses (Supporting Information, Figure S7) exhibited similar peak distributions. A complete list of identified compounds with associated retention times is provided in the Supporting Information, Tables S6 and S7. For cases where two different compounds overlapped in a single peak or an expected compound was not present in the NIST spectral library, assignments were made by analysis of the mass spectra (Supporting Information, Figures S8–S19). The visible peaks in Figure 5 correspond to alkanes, with branched isomers grouped by carbon content highlighted in gray blocks: C_8_H_18_ (RT < 2.4 min), C_9_H_20_ (RT 2.7–3.6 min), C_10_H_22_ (RT 3.8–5.6 min), C_11_H_24_ (RT 6–8.1 min), and C_12_H_26_ (RT 7.8–10.9 min). For both LDP and focused 30 fs ablation conditions, these alkanes comprised at least 95% of all identified GC products (Supporting Information, Table S8). Ablation with 4 ps pulses resulted in higher yields of alkenes and aromatic products as in previous work,^34^ with 91–92% alkane yields.

**Figure 6:**
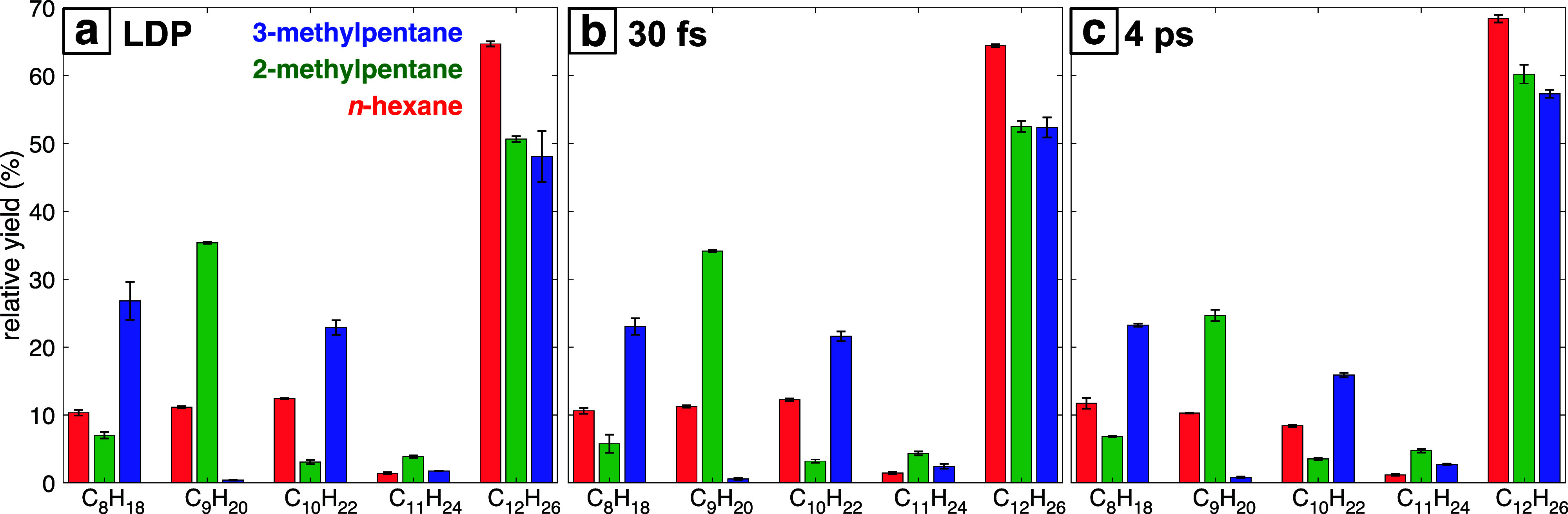
Relative yields of alkanes detected in GC: C_8_H_18_, C_9_H_20_, C_10_H_22_, C_11_H_24_, and C_12_H_26_ products obtained after laser ablation with (a) LDP conditions, (b) 30 fs pulses, and (c) 4 ps pulses. Error bars denote standard deviation over duplicate measurements.

As evident from Figure 5, each hexane isomer produces both different yields of alkanes according to molecular weight and different specific isomers. Only *n*-hexane produces the linear alkanes *n*-octane, *n*-nonane, and *n*-decane, as well as similar yields of C_8_, C_9_, and C_10_ branched alkanes. In contrast, 2-methylpentane produces mostly C_9_ branched alkanes, while 3-methylpentane produces almost exclusively C_8_ and C_10_ alkanes. All three hexane isomers produce numerous C_12_ alkanes, albeit distinct isomers. These patterns are quantified by the relative distributions of C_8_–C_12_ products obtained at each laser ablation condition reported in Figure 6 (tabulated values in Supporting Information, Table S9). The different laser ablation conditions do not significantly impact the product distribution selectivity, although somewhat higher relative yields of C_12_ alkanes are observed for picosecond ablation. The origins of the observed isomer-specific selectivity in the C_8_–C_12_ products and the reaction pathways involved will be further discussed in Section 4.

## 4 Discussion

This section discusses how the present results provide new insights into understanding the product distributions from laser ablation of liquid-phase organic molecules. The observed isomer-specific product distributions enable rational assumptions about the transient organic radicals and ions present in the laser plasma and how they react with each parent solvent molecule. However, we note that these putative species cannot be directly detected with only *ex situ* product measurements. Future investigations using radical scavengers as in radiolysis studies^44,46^ or *in situ* optical spectroscopy of radicals and cations as used in previous laser ablation studies^37 −40^ could confirm or refute the conjectured reactions and processes described in this section. In Section 4.1, the observed selectivity in the distributions of reaction products by number of carbon atoms, along with the specific structures of intermediate (C_8_–C_10_) alkanes and C_12_ dimers obtained for each hexane isomer are explained. Section 4.2 assesses the relative contributions of ion/radical chemistry from the laser plasma and mechanochemical processes from the laser-induced shockwaves to product generation based on the distributions obtained under different laser ablation conditions (LDP and focused 30 fs or 4 ps pulses). Finally in Section 4.3, we compare the product distributions from laser ablation with those reported in previous radiolysis, plasma discharge, and pyrolysis studies.

### 4.1 Product Selectivity

Comparison of the fragmentation patterns observed in gas-phase SFI-MS (Figure 1) with the gaseous product yields from laser ablation in liquid (particularly for LDP, Figures 3 and 4a) reveals a strong similarity in the selectivity toward C_2_, C_3_, or C_4_ ablation products of each hexane isomer. Whereas *n*-hexane exhibits no strong preference among C_2_–C_4_ products, 2-methylpentane exhibits selectivity toward C_3_ products and 3-methylpentane exhibits selectivity toward C_2_ and C_4_ products. This selectivity is also evident in the distributions of C_8_–C_10_ alkane products (Figures 5 and 6). Compared to the roughly equal amounts of C_8_H_18_, C_9_H_20_, and C_10_H_22_ obtained from *n*-hexane, 2-methylpentane produces high yields of C_9_H_20_ (by addition of a C_3_ fragment to a parent molecule), whereas 3-methylpentane produces almost exclusively C_8_H_18_ and C_10_H_22_ (by addition of C_2_ or C_4_ fragments to a parent molecule, respectively). In both cases, the product distributions can be rationalized by the different favorable bond-cleavage pathways in 2-methylpentane and 3-methylpentane shown in Figure 2: 2-methylpentane preferentially breaks into C_3_H_7_^+^ and C_3_H_7_^•^, while 3-methylpentane preferentially breaks into C_4_H_9_^+^ and C_2_H_5_^•^. The same selectivity for C–C bond scission is also expected to operate for the neutral molecules based on the literature bond dissociation energies^62^ and the ability to form secondary carbon radicals.

**Figure 7:**
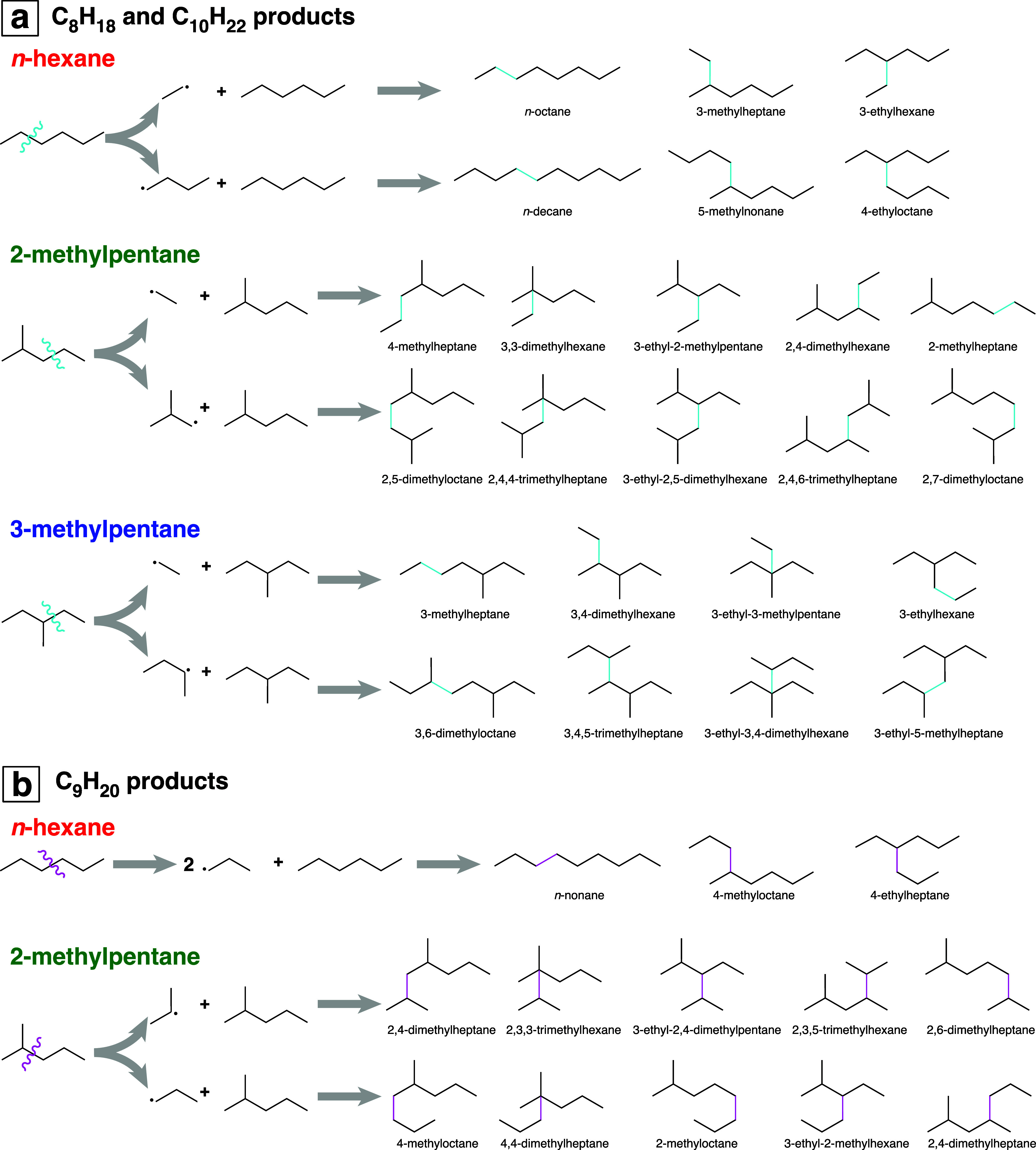
Mechanisms of (a) C_8_H_18_ and C_10_H_22_ formation and (b) C_9_H_20_ formation from addition of fragment radicals (without rearrangement) to parent hexane molecules. Only *n*-hexane and 2-methylpentane can break one bond to produce C_3_ radicals that form C_9_H_20_. Colors of squiggly lines and new bonds indicate initial cleavage into C_4_H_9_^•^ + C_2_H_5_^•^ (cyan) or two C_3_H_7_^•^ (magenta).

Beyond the relative yields of C_8_ through C_10_ products, the specific isomers observed indicate that almost all reactions occur between a parent hexane molecule and a radical fragment produced by one C–C bond scission, without rearrangement. Figure 7 shows the reaction pathways (ignoring H_2_ loss) that give rise to the C_8_ through C_10_ alkane structures comprising the intense peaks in Figure 5 (see also Supporting Information, Tables S6 and S10). In all three hexane isomers, C_8_H_18_ and C_10_H_22_ structures are formed by cleavage of a parent hexane molecule into C_2_H_5_^•^ and C_4_H_9_^•^, followed by addition to another parent hexane molecule (Figure 7a). Only *n*-hexane and 2-methylpentane can undergo a C–C bond scission to C_3_H_7_^•^ radicals that add to parent molecules to produce C_9_H_20_ products (Figure 7b). Across all hexane isomers and laser ablation conditions, at least 98% of C_8_ through C_10_ alkane yields observed in GC–MS from femtosecond ablation come from these direct bond-formation reactions between a parent hexane and fragment radical that does not undergo rearrangement or sequential C–C bond cleavage (94% for picosecond ablation, Supporting Information, Table S10). The low yields of reaction products from rearrangement or sequential fragmentation identified in Supporting Information, Table S7, are explained via the reaction pathways illustrated in Supporting Information, Figure S20. Nevertheless, the low probability of radical rearrangement or sequential bond scission indicates that ultrashort pulsed laser ablation involves primarily nonthermal reaction mechanisms that proceed over time scales faster than those of radical rearrangement. This feature distinguishes ultrashort laser ablation from plasma discharge, as will be discussed in Section 4.3.

**Figure 8:**
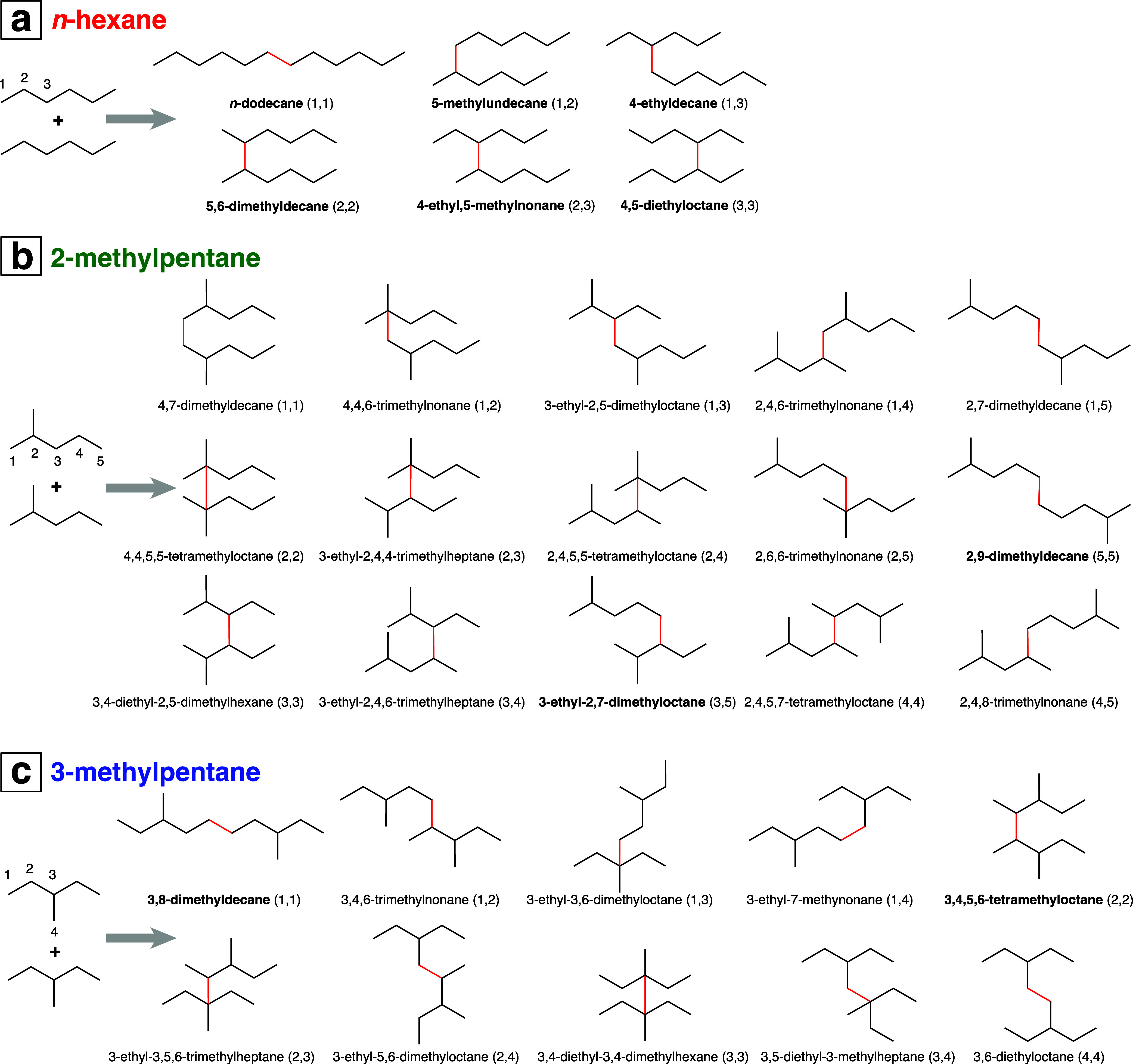
C_12_H_26_ isomers produced by direct dimerization of *n*-hexane (a), 2-methylpentane (b), and 3-methylpentane (c). Each newly created bond is highlighted in red. The numbers in parentheses after each product name denote the labeled carbon atoms between which a new bond is formed.

Further evidence for nonthermal reaction chemistry during laser ablation in liquid comes from the different structural isomers of C_12_H_26_ dimers produced from each hexane isomer. Previous γ radiolysis^43−46^ and pulsed laser ablation^31^ studies identified six C_12_H_26_ isomers produced from *n*-hexane: 4,5-diethyloctane; 4-ethyl-5-methylnonane; 5,6-dimethyldecane; 4-ethyldecane, 5-methylundecane, and *n*-dodecane. These structures arise by direct C–C bond formation between two *n*-hexane molecules without rearrangement, accompanied by H_2_ loss, as pointed out in ref (31). The same six dimer structures from *n*-hexane are observed in the present work (Supporting Information, Table S6). Figure 8a shows the formation mechanisms of each *n*-hexane dimer (ignoring the H_2_ loss), with the newly formed C–C bond highlighted in red. The numbers in parentheses denote the two carbon atoms between which the new bond is formed, according to the given labels. For instance, the creation of a bond between carbon 1 of the first *n*-hexane and carbon 2 of the second *n*-hexane to produce 5-methylundecane is denoted as (1,2). The analogous carbon–carbon bond-forming reactions in 2-methylpentane and 3-methylpentane form the dimer structures shown in Figure 8b,c, respectively. The dimer structures identified in GC–MS by matches to the NIST spectral data library are labeled in bold text in Figure 8. Other structures are not present in the NIST spectral data library. Nevertheless, number of distinct dimer structures predicted from Figure 8 is consistent with the number of observed peaks (excluding diastereomers) in the GC–MS measurements: 15 peaks are observed in 2-methylpentane and 10 peaks are observed in 3-methylpentane (Supporting Information, Table S6).

### 4.2 Ion/Radical Chemistry and Mechanochemistry Contributions

The observed isomer-specific selectivity for dimers formed by ablation of organic liquids with femtosecond laser pulses has recently been attributed to mechanochemical bond formation between solvent molecules due to compression by laser-induced shock waves.^31−33^ The experimental conditions used in this work allow for direct assessment of the hypothesis that shock wave-induced mechanochemistry, rather than plasma chemistry involving ions and radicals, acts as the primary driver to form molecular dimers for two reasons. First, the products arising solely from plasma chemistry can be determined by ablation under LDP, which eliminates shock waves and cavitation bubbles.^36,53^ Second, the comparison of product distributions obtained by (focused) femtosecond and picosecond ablation allows for determination of mechanochemical contributions to different product classes because the conversion efficiency of absorbed laser energy to mechanical energy in shock waves and cavitation bubbles increases with pulse width.^60^

From the GC–MS product distributions reported in Figure 6 and Supporting Information Table S9, it is evident that the relative yield of C_12_H_26_ dimers among all alkane products barely changes when comparing ablation with LDP and focused 30 fs pulses: *n*-hexane produces nearly identical dimer yields (64%), and the slight increases for 2-methylpentane (from 51% to 53%) and 3-methylpentane (from 48% to 52%) are within the reported errors. In contrast, the dimer yields significantly increase in each isomer for ablation with 4 ps pulses (68%) in *n*-hexane, 60% in 2-methylpentane, and 57% in 3-methylpentane. Moreover, the greater relative increase of dimer yields from 4 ps ablation compared to LDP in 2-methylpentane (15%) and 3-methylpentane (16%) compared to the 6% increase for *n*-hexane suggest a greater role for mechanochemical bond formation from the branched hexane isomers. However, it should be noted that the greater relative yield of C_12_H_26_ dimers in these molecules for 4 ps ablation could also be due to enhanced decomposition of the intermediate C_8_ through C_10_ alkanes during cavitation bubble collapse.

In contrast to the modest enhancement of dimer yields, the distributions of product gases and unsaturated liquid hydrocarbons undergo significant changes when comparing LDP and focused 30 fs ablation to 4 ps ablation, suggesting a greater role for mechanochemistry in these reactions. Compared to the substantial isomer-specific selectivity of the gaseous products seen in Figures 3 and 4a for LDP, all three hexane isomers produce predominantly C_2_H_2_ and C_2_H_4_ from 4 ps ablation (Figure 4c and Supporting Information, Figure S4 and Tables S1–S3). Similar high yields of C_2_H_2_ and C_2_H_4_ were also observed upon 4 ps ablation of other classes of organic liquids (ethanol, acetone, and toluene) in our previous work.^34^ The 5-fold enhancement in relative yields of alkene and aromatic products upon 4 ps ablation compared to LDP (Supporting Information, Figure S7 and Table S8) is also consistent with those previous results.^34^ Hence, we can conclude that mechanochemical processes driven by laser-induced shock waves and cavitation bubble collapse play a much more significant role in product formation upon ablation with picosecond pulses compared to femtosecond pulses, likely due to the inefficient conversion of laser energy to mechanical energy in femtosecond ablation as compared to longer pulse durations.^60^ We also note that the enhancement of gas and alkene formation in picosecond ablation was correlated with the generation of solid carbon products.^34^ Therefore, reactive species produced by mechanochemical processes are also likely to produce the amorphous or graphitic carbon shells observed around LSPC-generated nanoparticles produced with nanosecond laser pulses,^13−16^ although further studies using transient spectroscopy measurements would be needed to assess this hypothesis.

### 4.3 Comparing Laser Ablation in Liquid to Other Processing Methods

As discussed in the Introduction, product distributions from laser ablation of organic liquids have often been explained by analogy to gas-phase pyrolysis reactions^22,23,35,41,42^ or ion/radical reactions analogous to γ radiolysis or plasma discharge.^24^ In this section, we compare the observed product distributions from this work with previous reports from pyrolysis, plasma discharge, and γ radiolysis of hexanes to assess the relevance of each technique to understanding reaction pathways for ultrashort pulsed laser ablation.

The distributions of gas products observed in this work exhibit both similarities and differences with the product distributions from gas-phase pyrolysis, plasma discharge, and γ radiolysis. Pyrolysis of hexane isomers yields qualitatively similar selectivity in the distribution of C_2_–C_4_ products as observed in LDP (c.f., Figure 4a): similar yields of C_2_–C_4_ are observed in *n*-hexane, whereas more C_3_ is produced from 2-methylpentane compared to more C_2_ and C_4_ from 3-methylpentane.^49,50^ The same gas product selectivity trends are also observed in γ radiolysis.^43−48^ The gas products of microsecond pulsed plasma discharge of *n*-hexane, in contrast, more closely resemble the distribution from picosecond laser ablation (Figure 4c), with product yields trending as C_2_ > C_3_ > C_4_.^52^ It is also worth noting that the high yields of alkynes (C_2_H_2_, C_3_H_4_, C_4_H_2_, C_4_H_4_) observed in picosecond laser ablation (Supporting Information, Figure S4 and Tables S1–S3) are distinct from all other literature methods: all pyrolysis studies and most radiolysis studies do not report any alkynes, while only very low yields of alkynes relative to other hydrocarbons were reported in one radiolysis study^45^ and in pulsed plasma discharge.^52^

The distributions of liquid alkane products observed in ultrashort pulsed laser ablation are most similar to those produced by γ radiolysis. The same set of C_8_H_18_, C_9_H_20_, C_10_H_22_, C_11_H_24_, and C_12_H_26_ isomers reported in radiolysis^44−46^ were observed in this work. Some of the C_8_H_18_ and C_10_H_22_ isomers observed from 3-methylpentane in this work were also detected in radiolysis^48^ (no structural information on liquid products has been reported for radiolysis of 2-methylpentane to our knowledge). Hence, the laser-induced plasma reactions involving ions and radicals appear to strongly resemble those occurring under radiolysis conditions. Moreover, significant suppression of C_7_H_17_–C_12_H_26_ alkane formation when using radical scavengers was observed in radiolysis.^44,46^ Those results further support the direct radical addition mechanisms for producing C_8_H_18_–C_10_H_22_ alkanes proposed for laser ablation in Figure 7 and suggest that C_12_H_26_ dimers can be formed from combination of C_6_ radicals. Future investigations of laser ablation with radical scavengers could confirm these proposed addition mechanisms.

In contrast to the very similar liquid product distributions observed in γ radiolysis and laser ablation (particularly for LDP), the limited available studies of plasma discharge report completely different liquid product distributions from those observed in our work.^51,52^ Microwave plasma discharge in liquid *n*-hexane produced no alkanes, but instead the aromatic products styrene, phenylacetylene, naphthalene, and biphenylene.^51^ The aromatics toluene, styrene, phenylacetylene, and naphthalene were also observed in microsecond pulsed dielectric barrier discharge plasma processing of *n*-hexane.^52^ These aromatics were observed in only minute quantities from LDP and focused femtosecond ablation. Although their relative yields increased by more than an order of magnitude for picosecond ablation, they only constituted 1.5% of the total liquid products observed in GC–MS under this condition (Supporting Information, Table S8). Microsecond pulsed dielectric barrier discharge plasma processing reported significantly more unique liquid products in GC–MS compared to our work, although those products included alcohols that were produced due to processing under ambient conditions.^52^ That study observed a general decrease in product yields as C_8_ > C_9_ > C_10_ ≈ C_12_, compared to the significantly higher yields of C_12_ in our work (Figure 6). Although they reported four of the six C_12_H_26_ isomers in Figure 8 and six of the nine C_8_H_18_–C_10_H_22_ isomers in Figure 7, they also reported the C_8_H_18_ and C_9_H_20_ isomers that arise from rearrangements of C_3_H_7_^•^ and C_4_H_9_^•^ fragment radicals (see Supporting Information, Figure S20) in much higher yields than the trace amounts detected in our work, along with other higher molecular weight branched alkanes that would require radical rearrangements to form.^52^ These differences suggest that the much shorter plasma lifetime in ultrashort pulsed laser ablation (∼1 ns or less) compared to the duration of microsecond discharge plasma may enable the high selectivity of product isomers.

## 5 Conclusion

This work demonstrated that ultrashort pulsed laser ablation of organic liquids yields selective product distributions that are determined by the molecular structure of the starting material. The product distributions obtained from three hexane isomers indicated that the relative favorability of different C–C bond scission pathways determines the molecular weight distributions of both gas and liquid ablation products. The similar yields of C_2_–C_4_ and C_8_–C_10_ products in *n*-hexane is consistent with the lack of strong selectivity between scission of the two interior C–C bonds. In contrast, the selectivity toward C_3_ and C_9_ products in 2-methylpentane or C_2_, C_4_, C_8_, and C_10_ products in 3-methylpentane is consistent with a preference toward breaking the C–C bond that produces a secondary carbon radical or cation. Moreover, the high degree of isomer selectivity in the C_8_–C_12_ products indicates that radical rearrangements and sequential bond scission are rare. This result suggests that the reaction time scales during ultrashort pulsed laser ablation in liquid are faster than radical rearrangement time scales. Comparison of the product distributions obtained from LDP with focused 30 fs or 4 ps pulses demonstrated that the isomeric selectivity in liquid alkane products predominantly arises from the ion/radical reactions generated in the laser plasma, with mechanochemical effects of laser shock waves playing a minor role except for picosecond laser ablation. Comparison of the product distributions from ultrashort pulsed laser ablation in liquid with other high-energy processing methods showed that γ radiolysis generates the most similar product distributions and isomer selectivity, although some reaction pathways are also shared with plasma discharge and pyrolysis. Overall, the strong influence of molecular structure in the initial liquid on the distribution of laser ablation products suggests that careful consideration of the liquid medium should be taken when designing synthetic procedures for nanoparticle production by laser ablation in organic liquids.

## Supporting Information Available

Additional SFI mass spectra, GC chromatograms, and GC mass spectra; tabulated yields of products from SFI-MS and GC–MS measurements (PDF)
