# Supplementary material for: Understanding Selectivity in Product Distributions from Laser Ablation of Organic Liquids
Source: J Phys Chem B. 2024 Oct 16;128(42):10481–91. doi: 10.1021/acs.jpcb.4c05638 (PMC11514026; doi:10.1021/acs.jpcb.4c05638)
Supplement: Supplementary file 1 — jp4c05638_si_001.pdf [file jp4c05638_si_001.pdf]

# Supporting Information: Understanding Selectivity in Product Distributions from Laser Ablation of Organic Liquids

Samuel Harris, Ella Kaplan, Michael Aftel, and Katharine Moore Tibbetts*

*Department of Chemistry, Virginia Commonwealth University, Richmond, VA 23284, US*

E-mail: kmtibbetts@vcu.edu

## Contents

Number of pages: 29

Number of figures: 20

Number of tables: 10

# SI Supplemental SFI-MS data

All SFI-MS measurements were performed on duplicate ablation measurements for a given experiment (source molecule, laser condition: LDP, focused fs, focused ps). The 1800 nm, 25 fs SFI pulse was set to a high intensity of approximately $1.8 \times 10^{14}$ W cm$^{-2}$ to ensure detection of any $H_2$ (IE: 15.43 eV)[1] produced. Representative mass spectra taken before (gray) and after (colors, red: *n*-hexane; green: 2-methylpentane; blue: 3-methylpentane) at each laser ablation condition are shown in Figures S1 – S3. Enhanced signals of various gas products are visible in the colored spectra taken after ablation. The yields of gas products were obtained by subtracting the spectrum taken before ablation from the spectrum taken after ablation to obtain the "difference" spectrum, as shown for the LDP condition in Figure 3 of the main work. Integrating over the peak areas of these "difference" spectra the relative yields of each product, although it must be noted that the SFI-MS technique likely under-reports the yields of molecules with high IE values like $H_2$ and $CH_4$, as discussed in our previous work.[2,3] The difference spectra for focused 30 fs and 4 ps pulses are shown Figure S4 for comparison to Figure 3 of the main work. Tabulated percent yields of gas ablation products (normalized to total gas product yield) are given in Tables S1 – S3.

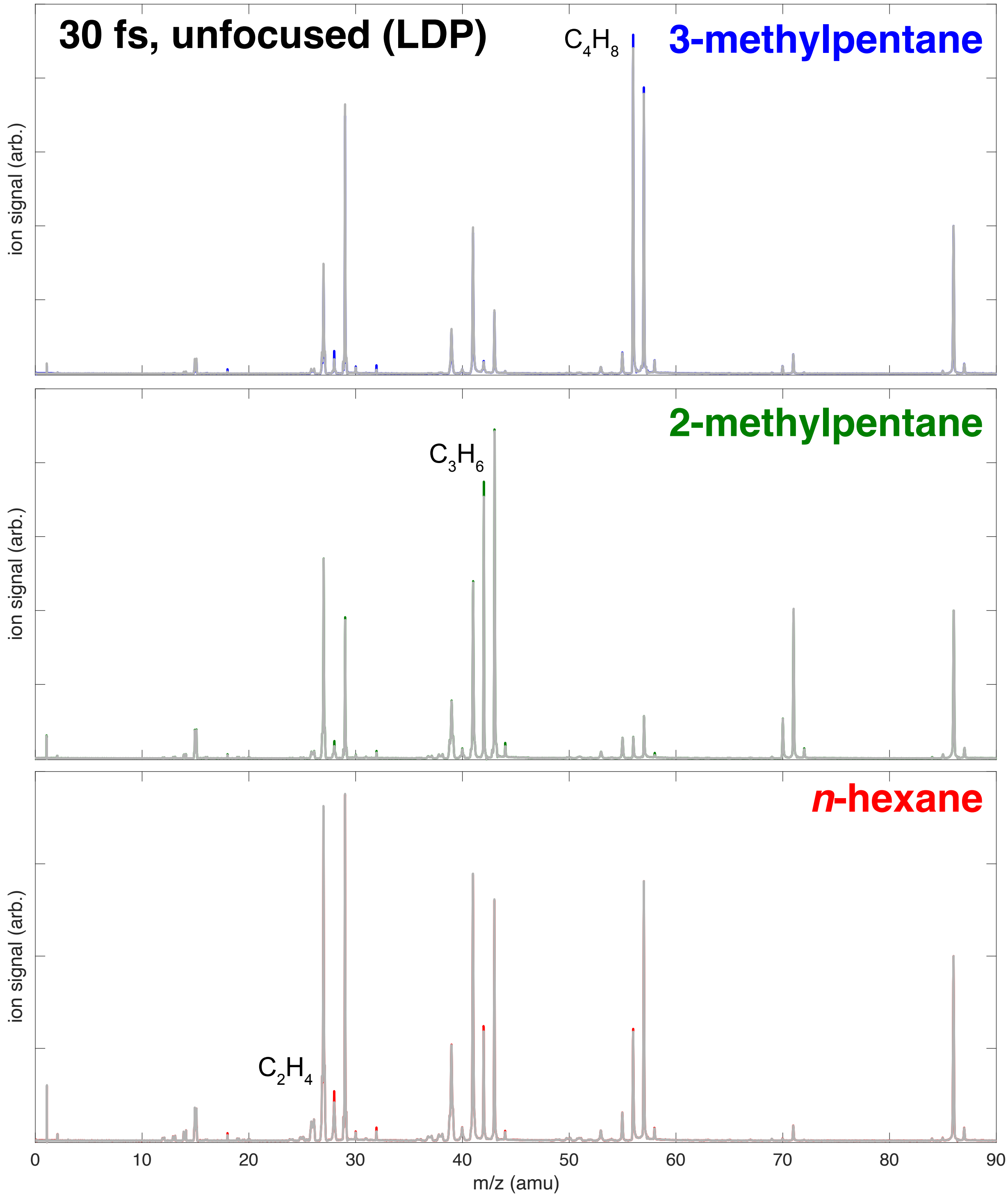


Figure S1: SFI-MS of *n*-hexane (red), 2-methylpentane (green), and 3-methylpentane (blue) before (grey) and after (colors) ablation with unfocused 30 fs pulses. Small enhancements in ion signals of $C_2H_4$, $C_3H_6$, and $C_4H_8$ in the spectra measured after ablation are visible.

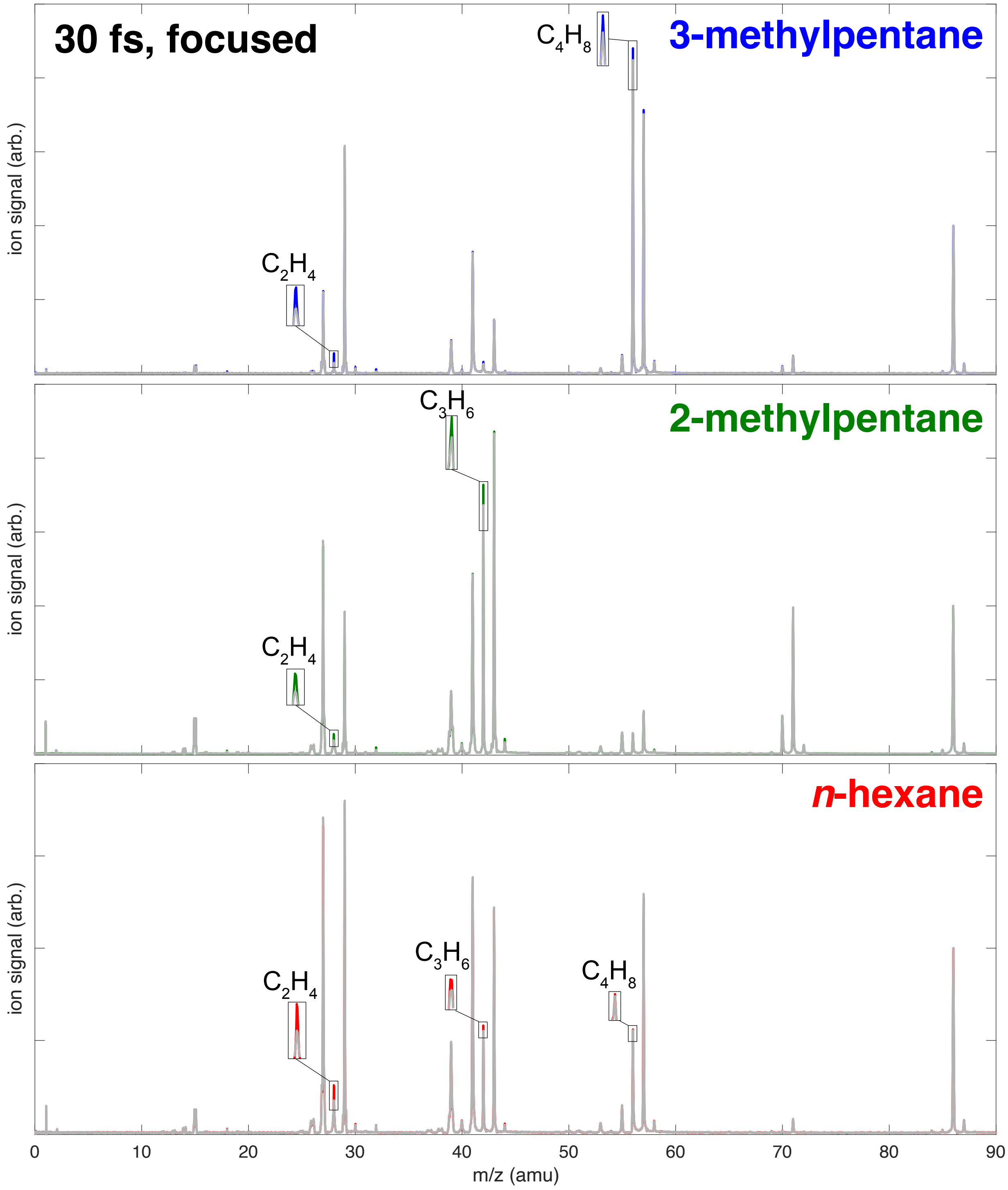


Figure S2: SFI-MS of *n*-hexane (red), 2-methylpentane (green), and 3-methylpentane (blue) before (grey) and after (colors) ablation with focused 30 fs pulses. The subtle enhancement of ion signals of $C_2H_4$, $C_3H_6$, and $C_4H_8$ in the spectra measured after ablation are magnified on each spectrum for clarity.

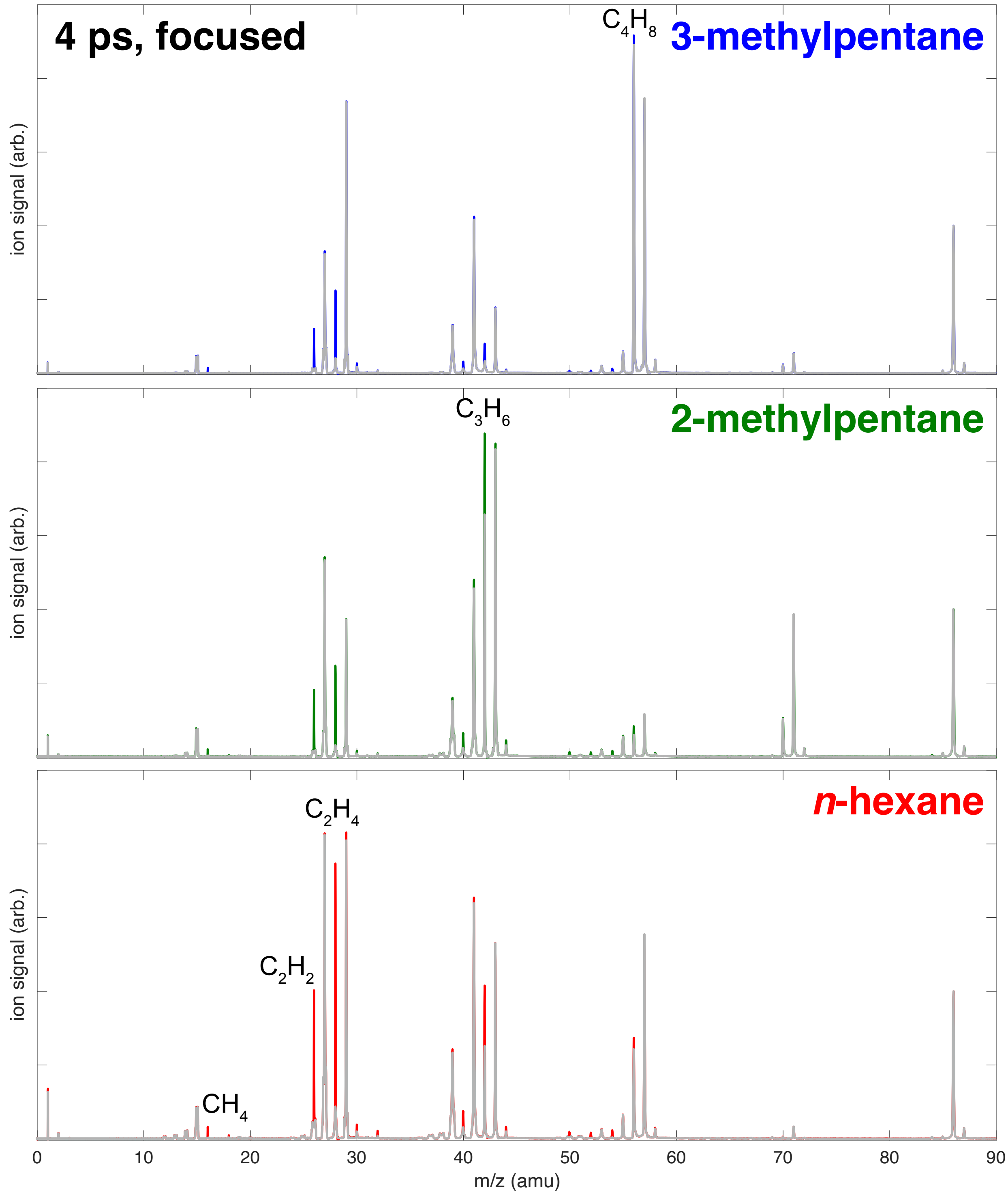


Figure S3: SFI-MS of *n*-hexane (red), 2-methylpentane (green), and 3-methylpentane (blue) before (grey) and after (colors) ablation with focused 4 ps pulses. The enhanced ion signals of $CH_4$, $C_2H_2$, $C_2H_4$, $C_3H_6$, and $C_4H_8$ in the spectra measured after ablation are indicated.

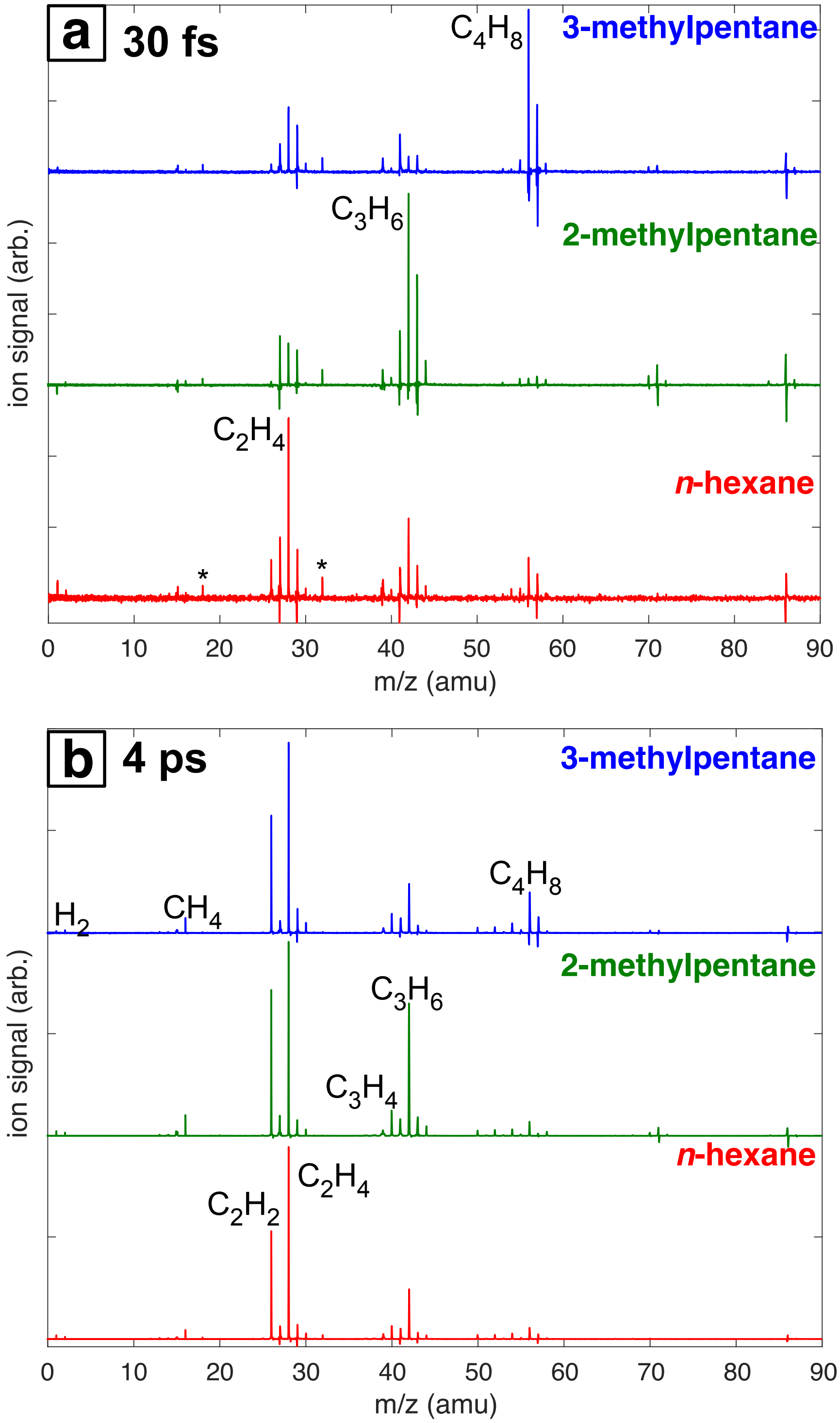


Figure S4: Difference mass spectra for each hexane isomer for focused fs (a) and ps (b) conditions.

Table S1: Relative yields of gas products in SFI-MS for *n*-hexane. Error bars indicate standard deviation from two separate experiments. IE values from NIST[1] are given for reference.

| species | IE (eV) | LDP | 30 fs | 4 ps |
|---|---|---|---|---|
| $H_2$ | 15.43 | 0.9±0.2 | 0.9±0.3 | 0.3±0.1 |
| $CH_4$ | 12.61 | 0.9±0.4 | 0.6±0.2 | 1.9±0.3 |
| $C_2H_2$ | 11.4 | 3.4±0.9 | 6±4 | 24±1 |
| $C_2H_4$ | 10.51 | 42±3 | 50±1 | 48±1 |
| $C_2H_6$ | 11.52 | 3±1 | 2.4±0.7 | 1.6±0.1 |
| $C_3H_4$ | 10.36 | 1.2±0.3 | 2.2±0.3 | 3.4±0.1 |
| $C_3H_6$ | 9.73 | 23±2 | 22±2 | 13±1 |
| $C_3H_8$ | 10.94 | 5.3±0.4 | 4±2 | 1.0±0.1 |
| $C_4H_2$ | 10.17 | | | 1.1±0.2 |
| $C_4H_4$ | ≤9.58 | | | 1.2±0.1 |
| $C_4H_6$ | ≤10.18 | 2±1 | 1.4±0.6 | 1.6±0.1 |
| $C_4H_8$ | ≤9.55 | 15±1 | 8.6±0.1 | 2.6±0.8 |
| $C_4H_{10}$ | ≤10.68 | 3.3±0.2 | 2.1±0.2 | 0.09±0.01 |
| $C_5H_6$ | | | | 0.12±0.03 |
| $C_5H_8$ | | | | 0.18±0.05 |
| $C_5H_{10}$ | | | | |
| $C_5H_{12}$ | | | | 0.08±0.07 |
| $C_6H_{12}$ | | | | |

Table S2: Relative yields of gas products in SFI-MS for 2-methylpentane. Error bars indicate standard deviation from two separate experiments. IE values from NIST[1] are given for reference.

| species | IE (eV) | LDP | 30 fs | 4 ps |
|---|---|---|---|---|
| $H_2$ | 15.43 | 0.5±0.1 | 0.8±0.3 | 0.3±0.1 |
| $CH_4$ | 12.61 | 1.0±0.2 | 1.3±0.1 | 2.1±0.5 |
| $C_2H_2$ | 11.4 | 0.7±0.5 | 2±1 | 20±3 |
| $C_2H_4$ | 10.51 | 13±4 | 20±3 | 27±2 |
| $C_2H_6$ | 11.52 | 0.5±0.3 | 1.2±0.1 | 1.0±0.1 |
| $C_3H_4$ | 10.36 | 2.2±0.5 | 3.5±0.2 | 5.2±0.4 |
| $C_3H_6$ | 9.73 | 55±5 | 50±2 | 32±3 |
| $C_3H_8$ | 10.94 | 9±2 | 9.5±0.5 | 2.1±0.1 |
| $C_4H_2$ | 10.17 | | | 1.2±0.3 |
| $C_4H_4$ | ≤9.58 | | | 1.3±0.3 |
| $C_4H_6$ | ≤10.18 | | | 1.6±0.4 |
| $C_4H_8$ | ≤9.55 | 2±1 | 2.4±0.8 | 3.3±0.9 |
| $C_4H_{10}$ | ≤10.68 | 7±1 | 2.3±0.4 | 0.6±0.3 |
| $C_5H_6$ | | | | 0.2±0.1 |
| $C_5H_8$ | | | | 0.2±0.1 |
| $C_5H_{10}$ | | 2.8±0.6 | 3.1±0.2 | 1.1±0.2 |
| $C_5H_{12}$ | | 4±2 | 1.6±0.5 | 0.3±0.1 |
| $C_6H_{12}$ | | 2±1 | 1.5±0.5 | 0.3±0.2 |

Table S3: Relative yields of gas products in SFI-MS for 2-methylpentane. Error bars indicate standard deviation from two separate experiments. IE values from NIST[1] are given for reference.

| species | IE (eV) | LDP | 30 fs | 4 ps |
|---|---|---|---|---|
| $H_2$ | 15.43 | 0.5±0.1 | 0.6±0.2 | 0.3±0.1 |
| $CH_4$ | 12.61 | 0.5±0.3 | 1.1±0.1 | 2.2±0.2 |
| $C_2H_2$ | 11.4 | 1.4±0.5 | 4.1±0.4 | 21±2 |
| $C_2H_4$ | 10.51 | 37±5 | 41±5 | 36±4 |
| $C_2H_6$ | 11.52 | 5.3±0.3 | 6.0±0.8 | 2.3±0.1 |
| $C_3H_4$ | 10.36 | 1.1±0.9 | 2.5±0.4 | 4.9±0.4 |
| $C_3H_6$ | 9.73 | 7.2±0.8 | 10.8±0.1 | 13±1 |
| $C_3H_8$ | 10.94 | 0.9±0.2 | 1.3±0.3 | 0.6±0.1 |
| $C_4H_2$ | 10.17 | | | 1.5±0.3 |
| $C_4H_4$ | ≤9.58 | | | 1.7±0.2 |
| $C_4H_6$ | ≤10.18 | 0.7±0.5 | 1.4±0.4 | 2.8±0.5 |
| $C_4H_8$ | ≤9.55 | 36±2 | 24±4 | 12±3 |
| $C_4H_{10}$ | ≤10.68 | 4.7±0.7 | 4.5±0.2 | 0.5±0.1 |
| $C_5H_6$ | | | | 0.14±0.07 |
| $C_5H_8$ | | | | 0.2±0.1 |
| $C_5H_{10}$ | | 3±1 | 2.6±0.7 | 0.9±0.4 |
| $C_5H_{12}$ | | | | |
| $C_6H_{12}$ | | 1.2±0.3 | | 0.4±0.3 |

# SII Supplemental GC-MS data

Integration of signals was conducted automatically using MSD ChemStation F.01.01.2317 software with MS Search utilizing the NIST17 library unless otherwise noted. In many cases, specific isomers of products could not be distinguished; in these cases only the chemical formulas are given. To assist with analysis of the laser ablation products, two additional sets of GC measurements were conducted (1) on the as-received liquids ,with added 400 $\mu$M acetophenone, to quantify the concentrations of any contaminant species; and (2) on an isoparaffin standard mixture.

## SII.1 As-received liquids

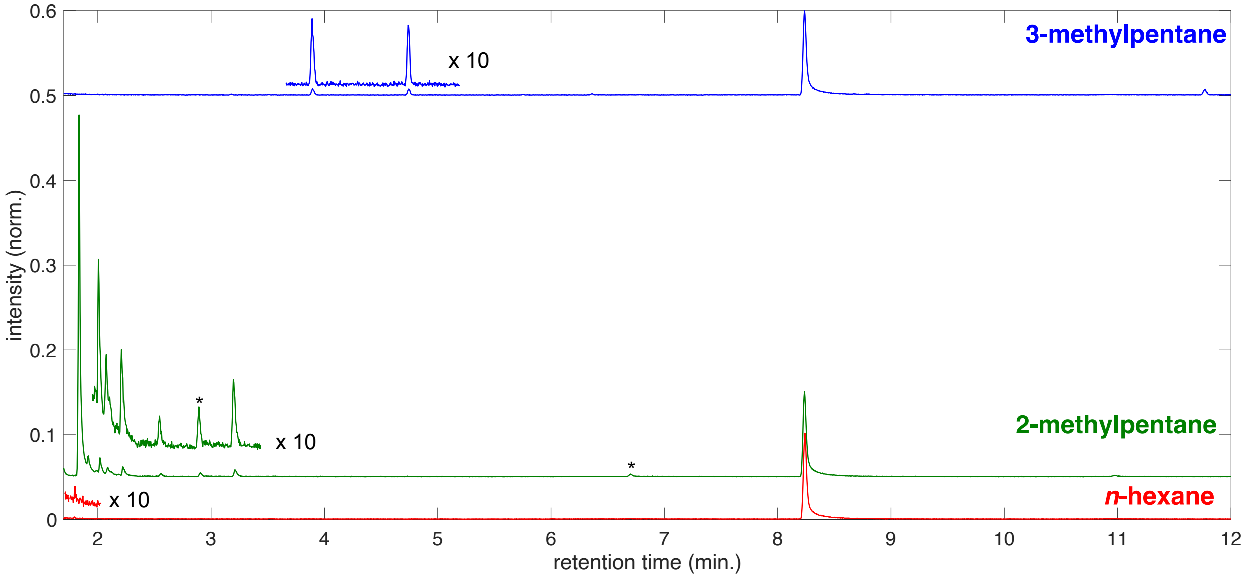


Figure S5: Chromatogram of as-received $n$-hexane, 2-methylpentane, and 3-methylpentane. Peaks in the 2-methylpentane spectrum marked with a $*$ are siloxanes from the column. Acetophenone (RT 8.24 min.) at concentration 400 $\mu$M is used as internal standard.

Table S4: Quantified contaminants (concentrations in mM) from as-received solvents based on integrated acetophenone signal.

| | | *n*-hexane | | | | 2-methylpentane | | | | 3-methylpentane | |
|---|---|---|---|---|---|---|---|---|---|---|---|
| **RT** | **mM** | **m/z** | **assignment** | **RT** | **mM** | **m/z** | **assignment** | **RT** | **mM** | **m/z** | **assignment** |
| 1.797 | 2.5 | 98, 83, 55, 41 | methylcyclohexane | 1.835 | 755 | 87, 69, 58, 45 | 2-methyl-2-pentanol | 3.895 | 19 | 120, 91, 77, 66 | $C_9H_{12}$ |
| | | | | 1.917 | 73 | 100, 85, 69, 58 | $C_6H_{10}O$ | 4.747 | 18 | 120, 105, 91, 77 | $C_9H_{12}$ |
| | | | | 2.024 | 49 | 100, 84, 71, 57 | $C_6H_{10}O$ | 11.774 | 20 | 168, 125, 111, 97 | $C_{12}H_{24}$ |
| | | | | 2.088 | 42 | 87, 69, 56, 45 | $C_6H_{12}O$ | | | | |
| | | | | 2.225 | 36 | 100, 84, 73, 59 | $C_6H_{10}O$ | | | | |
| | | | | 2.559 | 15 | 98, 83, 67, 55 | $C_6H_8O$ | | | | |
| | | | | 3.214 | 21 | 99, 83, 72, 55 | unknown | | | | |

## SII.2 Isoparaffin standard mixture

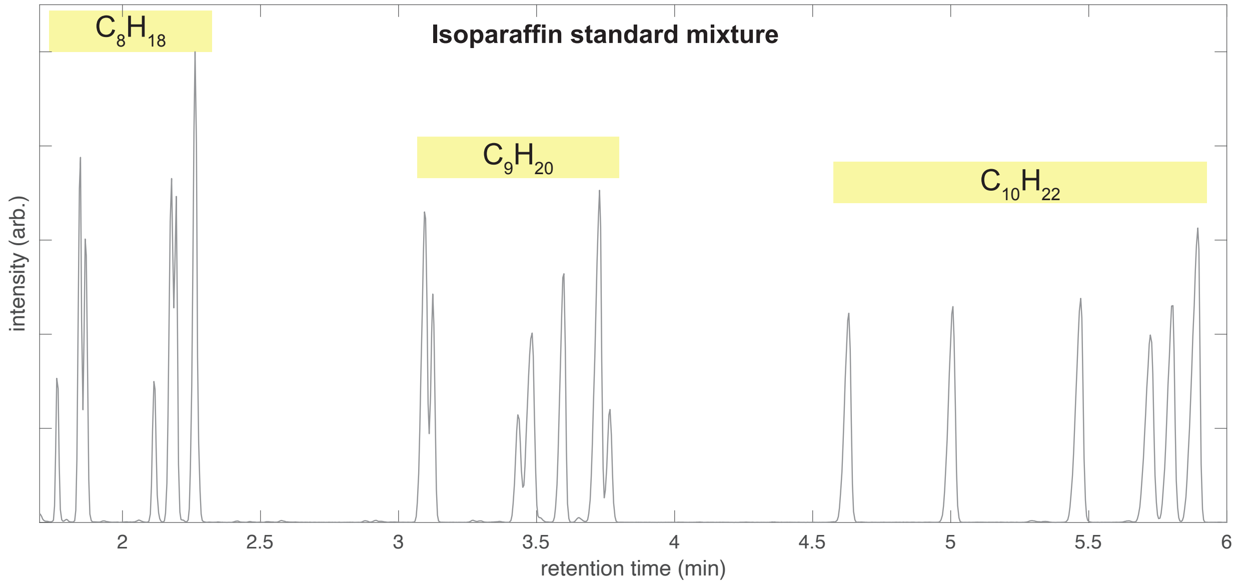


Figure S6: Chromatogram of isoparaffin standard mixture.

Table S5: Retention times for isoparaffins in Figure S6 and molecular assignments.

| RT (min) | assignment | RT (min) | assignment |
|---|---|---|---|
| 1.767 | 2,2-dimethylhexane | 3.484 | 3,4-dimethylheptane |
| 1.848 | 2,5-dimethylhexane | 3.599 | 2-methyloctane |
| 1.869 | 2,4-dimethylhexane<br>2,2,3-trimethylpentane | 3.728 | 3-methyloctane |
| 2.118 | 2,3-dimethylhexane | 3.766 | 3,3-diethylpentane |
| 2.178 | 2-methylheptane | 4.631 | 2,2-dimethyloctane |
| 2.195 | 4-methylheptane | 5.008 | 3,3-dimethyloctane |
| 2.263 | 3-methylheptane<br>3-ethylhexane | 5.471 | 2,3-dimethyloctane |
| 3.098 | 2,5-dimethylheptane | 5.723 | 2-methylnonane |
| 3.124 | 3,3-dimethylheptane<br>3,5-dimethylheptane | 5.805 | 3-ethyloctane |
| 3.437 | 2,3-dimethylheptane | 5.895 | 3-methylnonane |

## SII.3 Ablation product chromatograms and assignments

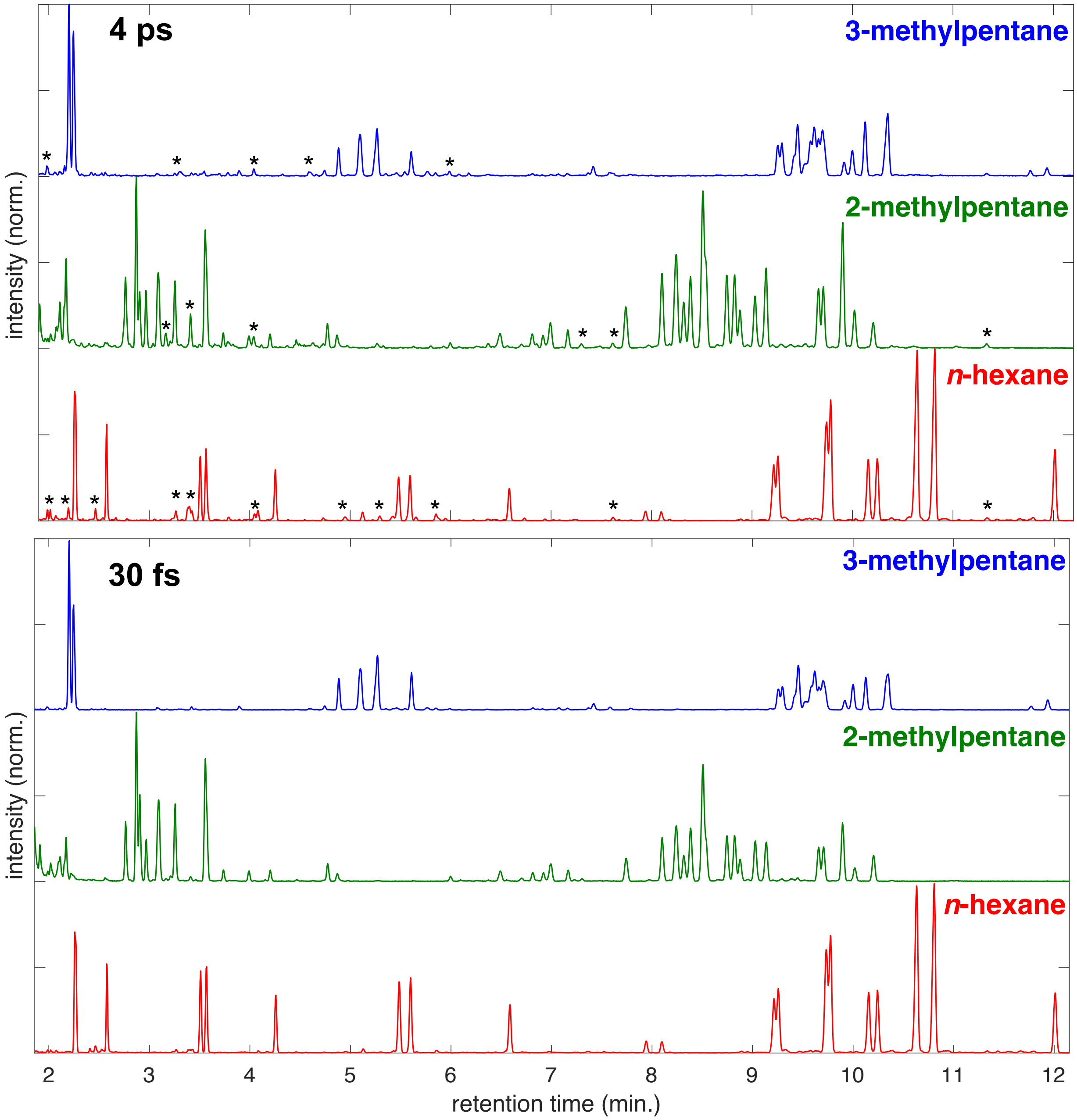


Figure S7: Chromatograms of $n$-hexane (red), 2-methylpentane (green), and 3-methylpentane (blue) ablated with focused 4 ps pulses (top) and 30 fs pulses (bottom). Enhancement of minor peaks assigned to alkenes and aromatics from 4 ps ablation are indicated with a $*$.

Table S6: Retention times for major ablation products in Figure S7 with molecular assignments. Specific molecules, color-coded by source liquid (red: *n*-hexane, green: 2-methylpentane, blue: 3-methylpentane) are assigned by matches to NIST library, with the following notes: $\ast$ denotes products that overlapped with a contaminant in the as-received 2-methylpentane (see Figures S8 and S9), ‡ denotes molecules not present in the NIST library (see Figures S14 and S15), and $\circ$ denotes diastereomers of the same molecule determined based on peak area and mass spectrum.

| RT (min) | assignment | m/z | RT (min) | assignment | m/z |
|---|---|---|---|---|---|
| 1.848 | 2,4-dimethylhexane* | 114, 99, 85, 71 | 7.937 | 4-ethylnonane | 156, 126, 112, 98 |
| 1.912 | 3,3-dimethylhexane* | 99, 85, 71, 57 | 8.096 | 5-methyldecane | 156, 98, 85, 71 |
| 2.114 | 3-ethyl,2-methylpentane | 114, 85, 70, 55 | 8.106 | $C_{12}H_{26}$ | 170, 155, 127, 113 |
| 2.174 | 2-methylheptane<br>4-methylheptane | 114, 99, 70, 57<br>114, 99, 85, 71 | 8.241 | $C_{12}H_{26}$ | 170, 155, 127, 113 |
| 2.208 | 3,4-dimethylhexane<br>3-ethyl,3-methylpentane | 114, 85, 69, 56<br>114, 99, 85, 69 | 8.318 | $C_{12}H_{26}$ | 127, 85, 84, 71 |
| 2.25 | 3-methylheptane<br>3-ethylhexane | 114, 85, 57, 43<br>114, 85, 71, 57 | 8.387 | $C_{12}H_{26}$ | 127, 99, 85, 71 |
| 2.58 | n-octane | 114, 85, 71, 57 | 8.511 | $C_{12}H_{26}$<br>$C_{12}H_{26}$ | 155, 127, 85, 71<br>170, 155, 12, 113 |
| 2.765 | 2,3,5-trimethylhexane | 128, 85, 71, 57 | 8.746 | $C_{12}H_{26}$ | 141, 127, 97, 85 |
| 2.872 | 2,4-dimethylheptane | 128, 85, 71, 57 | 8.824 | $C_{12}H_{26}$° | 141, 127, 126, 97 |
| 2.906 | 4,4-dimethylheptane | 113, 85, 71, 57 | 8.879 | $C_{12}H_{26}$° | 141, 127, 126, 97 |
| 2.97 | 2,6-dimethylheptane | 128, 113, 85, 71 | 9.029 | $C_{12}H_{26}$ | 127, 97, 85, 71 |
| 3.094 | 3-ethyl-2,4-dimethylpentane<br>2,3,3-trimethylhexane | 113, 85, 69, 57 | 9.136 | 3-ethyl-2,7-dimethyloctane | 141, 126, 111, 98 |
| 3.257 | 3-ethyl-2-methylhexane | 128, 99, 84, 69 | 9.209 | 4,5-diethyloctane° | 141, 127, 99, 84 |
| 3.505 | 4-ethylheptane | 128, 98, 85, 69 | 9.252 | 4,5-diethyloctane° | 141, 127, 99, 84 |
| 3.565 | 4-methyloctane<br>2-methyloctane | 128, 98, 85, 71<br>128, 113, 85, 71 | 9.26 | $C_{12}H_{26}$° | 170, 142, 112, 99 |
| 3.737 | 2,4,6-trimethylheptane | 142, 127, 99, 85 | 9.303 | $C_{12}H_{26}$° | 170, 142, 112, 99 |
| 3.989 | 2,4,4-trimethylheptane‡ | 127, 99, 85, 71 | 9.419 | 3,4,5,6-tetramethyloctane° | 113, 85, 71, 57 |
| 4.203 | 3-ethyl-2,5-dimethylhexane | 142, 113, 98, 85 | 9.461 | $C_{12}H_{26}$ | 141, 113, 99, 85 |
| 4.255 | n-nonane | 128, 99, 85, 71 | 9.53 | 3,4,5,6-tetramethyloctane° | 113, 85, 71, 57 |
| 4.773 | 2,5-dimethyloctane | 142, 127, 113, 99 | 9.581 | $C_{12}H_{26}$ | 168, 141, 112, 99 |
| 4.871 | 2,7-dimethyloctane | 127, 99, 85, 71 | 9.624 | $C_{12}H_{26}$ | 170, 141, 112, 99 |
| 4.888 | 3-ethyl-5-methyl heptane | 142, 113, 85, 71 | 9.659 | $C_{12}H_{26}$° | 170, 155, 141, 127 |
| 5.102 | 3,6-dimethyloctane | 142, 113, 85, 71 | 9.671 | $C_{12}H_{26}$° | 170, 141, 113, 99 |
| 5.273 | 3,4,5-trimethylheptane | 142, 85, 69, 57 | 9.700 | $C_{12}H_{26}$° | 170, 155, 141, 127 |
| 5.484 | 4-ethyloctane | 142, 112, 98, 85 | 9.710 | $C_{12}H_{26}$° | 170, 141, 113, 99 |
| 5.595 | 5-methylnonane | 142, 112, 85, 71 | 9.727 | 4-ethyl-5-methylnonane° | 170, 141, 113, 99 |
| 5.608 | 3-ethyl-3,4-dimethylhexane‡ | 140, 127, 113, 85 | 9.766 | 4-ethyl-5-methylnonane° | 170, 141, 113, 99 |
| 5.997 | $C_{11}H_{24}$ | 141, 113, 99, 85 | 9.898 | $C_{12}H_{26}$ | 170, 155, 127, 111 |
| 6.49 | $C_{11}H_{24}$ | 156, 112, 99, 85 | 9.919 | $C_{12}H_{26}$ | 170, 141, 111, 99 |
| 6.584 | n-decane | 142, 113, 99, 85 | 9.997 | $C_{12}H_{26}$° | 141, 85, 71, 57 |
| 6.811 | $C_{11}H_{24}$ | 156, 113, 85, 71 | 10.018 | 2,9-dimethyldecane | 170, 155, 127, 113 |
| 6.815 | $C_{11}H_{24}$ | 127, 113, 98, 85 | 10.133 | $C_{12}H_{26}$ | 170, 141, 111, 99 |
| 6.918 | $C_{11}H_{24}$ | 156, 113, 85, 71 | 10.147 | 5,6-dimethyldecane° | 170, 113, 84, 71 |
| 6.991 | $C_{11}H_{24}$ | 156, 127, 113, 85 | 10.202 | $C_{12}H_{26}$ | 127, 99, 85, 71 |
| 7.162 | $C_{11}H_{24}$ | 156, 127, 97, 85 | 10.237 | 5,6-dimethyldecane° | 170, 113, 84, 71 |
| 7.166 | $C_{11}H_{24}$ | 156, 141, 113, 85 | 10.352 | $C_{12}H_{26}$°<br>3,8-dimethyldecane | 141, 85, 71, 57<br>155, 141, 113, 99 |
| 7.304 | $C_{11}H_{24}$ | 156, 141, 127, 113 | 10.626 | 4-ethyldecane | 170, 140, 126, 99 |
| 7.423 | $C_{11}H_{24}$ | 156, 127, 98, 85 | 10.798 | 5-methylundecane | 170, 155, 140, 112 |
| 7.586 | $C_{11}H_{24}$ | 156, 127, 97, 84 | 12.005 | n-dodecane | 170, 141, 127, 112 |
| 7.745 | $C_{12}H_{26}$ | 170, 113, 99, 85 | | | |

Table S7: Retention times for minor ablation products in Figure S7 with molecular assignments. Specific molecules are assigned by matches to NIST library where possible; otherwise products are classified as alkenes (cyan), aromatics (magenta), or alkanes (dark red). Source molecules (*n*-hexane: nHex; 2-methylpentane: 2MP; 3-methylpentane: 3MP) indicated.

| RT (min) | assignment | m/z | samples | RT (min) | assignment | m/z | samples |
|---|---|---|---|---|---|---|---|
| 1.968 | $C_8H_{16}$ alkene | 112, 97, 83, 69 | 2MP | 5.123 | $C_{10}H_{20}$ alkene | 140, 113, 98, 84 | nHex |
| 1.985 | $C_8H_{16}$ alkene | 112, 83, 70, 55 | 3MP | 5.269 | $C_{10}H_{20}$ alkene | 140, 84, 69, 58 | 2MP |
| 1.989 | $C_8H_{16}$ alkene | 112, 83, 70, 55 | nHex | 5.295 | $C_{10}H_{20}$ alkene | 140, 111, 98, 83 | nHex |
| 1.994 | $C_8H_{16}$ alkene | 112, 94, 84, 69 | 2MP | 5.329 | $C_{10}H_{20}$ alkene | 140, 112, 83, 69 | 2MP |
| 2.015 | $C_8H_{16}$ alkene | 112, 83, 70, 55 | nHex | 5.355 | $C_{10}H_{20}$ alkene | 140, 111, 85, 69 | 3MP |
| 2.07 | $C_8H_{16}$ alkene | 112, 83, 55, 41 | nHex | 5.428 | 3,4-dimethyloctane | 142, 113, 84, 71 | nHex |
| 2.075 | $C_8H_{16}$ alkene | 112, 97, 84, 70 | 2MP | 5.462 | $C_{10}H_{20}$ alkene | 140, 113, 85, 69 | 3MP |
| 2.157 | $C_8H_{16}$ alkene | 112, 83, 70, 55 | 3MP | 5.544 | $C_{10}H_{20}$ alkene | 140, 111, 98, 85 | 3MP |
| 2.199 | toluene | 92, 91, 65, 51 | nHex 2MP 3MP | 5.633 | $C_{10}H_{22}$ branched alkane | 142, 112, 98, 71 | 2MP |
| 2.328 | $C_8H_{16}$ alkene | 112, 94, 84, 69 | 2MP | 5.659 | $C_{10}H_{20}$ alkene | 140, 98, 84, 70 | nHex |
| 2.405 | $C_8H_{16}$ alkene | 112, 95, 69, 55 | 2MP | 5.775 | $C_{10}H_{20}$ alkene | 140, 112, 85, 69 | 3MP |
| 2.426 | $C_8H_{16}$ alkene | 112, 83, 70, 56 | 2MP | 5.805 | $C_{10}H_{20}$ alkene | 140, 112, 97, 84 | 2MP |
| 2.465 | $C_8H_{16}$ alkene | 112, 83, 68, 55 | 2MP | 5.852 | $C_{10}H_{20}$ alkene | 140, 112, 85, 71 | 3MP |
| 2.469 | 1-octene | 112, 83, 70, 55 | nHex | 5.856 | 3-methylnonane | 142, 112, 71, 57 | nHex |
| 2.529 | $C_8H_{16}$ alkene | 112, 83, 69, 55 | 3MP | 5.933 | $C_{10}H_{20}$ alkene | 140, 115, 98, 84 | 2MP |
| 2.541 | $C_8H_{16}$ alkene | 112, 83, 70, 55 | nHex | 5.95 | $C_{10}H_{20}$ alkene | 140, 98, 85, 69 | nHex |
| 2.563 | $C_8H_{16}$ alkene | 112, 83, 70, 55 | 3MP | 5.989 | $C_{10}H_{20}$ alkene | 140, 111, 83, 70 | 3MP |
| 2.585 | $C_8H_{16}$ alkene | 112, 95, 83, 70 | 2MP | 6.182 | $C_{10}H_{20}$ alkene | 140, 111, 83, 70 | 3MP |
| 2.67 | $C_8H_{16}$ alkene | 112, 83, 70, 55 | nHex | 6.374 | $C_9H_{10}$ aromatic | 118, 103, 91 | 2MP |
| 2.781 | $C_8H_{16}$ alkene | 112, 83, 70, 55 | nHex | 6.567 | $C_{11}H_{22}$ alkene | 154, 111, 98, 85 | 2MP |
| 3.039 | $C_9H_{18}$ alkene | 126, 84, 69, 55 | 2MP | 6.738 | 4-decene | 140, 111, 97, 84 | nHex |
| 3.077 | 2,5-dimethylheptane | 128, 99, 71, 57 | 3MP | 6.858 | $C_{11}H_{22}$ alkene | 154, 112, 96, 85 | 2MP |
| 3.167 | $C_9H_{18}$ alkene | 126, 85, 69, 55 | 2MP | 6.939 | 2-decene | 140, 111, 97, 84 | nHex |
| 3.231 | $C_9H_{18}$ alkene | 126, 97, 83, 55 | nHex | 7.616 | $C_9H_8$ aromatic | 116, 115, 89 | nHex 2MP 3MP |
| 3.269 | 3-ethyl-2-methylhexane | 128, 88, 84, 69 | nHex | 7.971 | $C_{12}H_{24}$ alkene | 168, 134, 112, 83 | 2MP |
| 3.308 | $C_9H_{18}$ alkene | 126, 97, 85, 69 | 3MP | 8.661 | $C_{12}H_{24}$ alkene | 168, 130, 115, 83 | 2MP |
| 3.402 | $C_9H_{18}$ alkene | 126, 84, 69, 55 | nHex | 8.938 | $C_{12}H_{24}$ alkene | 168, 126, 84, 69 | 3MP |
| 3.411 | $C_9H_{18}$ alkene | 126, 111, 87, 84 | 2MP | 8.956 | $C_{12}H_{24}$ alkene | 168, 126, 112, 85 | 2MP |
| 3.424 | $C_9H_{18}$ alkene | 126, 99, 70, 57 | 3MP | 9.042 | $C_{12}H_{24}$ alkene | 168, 128, 111, 84 | 3MP |
| 3.428 | 2,3-dimethylheptane | 128, 84, 71, 57 | nHex | 9.205 | $C_{12}H_{24}$ alkene | 168, 141, 127, 113 | 2MP |
| 3.463 | 3,4-dimethylheptane | 128, 99, 85, 70 | 3MP | 9.286 | $C_{12}H_{24}$ alkene | 168, 141, 112, 85 | 2MP |
| 3.505 | $C_8H_{10}$ aromatic | 106, 91, 77 | 2MP | 9.384 | $C_{12}H_{24}$ alkene | 168, 141, 99, 84 | 2MP |
| 3.548 | 6-methyl-1-octene | 128, 97, 83, 70 | 3MP | 9.534 | $C_{12}H_{24}$ alkene | 168, 128, 97, 85 | 2MP |
| 3.655 | $C_8H_{10}$ aromatic | 106, 91, 77 | 2MP | 10.035 | $C_{12}H_{24}$ alkene | 168, 139, 111, 98 | nHex |
| 3.698 | 3-methyloctane | 128, 98, 71, 57 | 3MP | 10.254 | $C_{12}H_{24}$ alkene | 168, 128, 111, 98 | 3MP |
| 3.792 | phenylacetylene | 102, 76, 63 | nHex 2MP 3MP | 10.39 | $C_{12}H_{24}$ alkene | 168, 125, 111, 98 | nHex |
| 3.835 | $C_9H_{18}$ alkene | 126, 98, 84, 70 | 2MP | 10.391 | $C_{12}H_{24}$ alkene | 168, 129, 97, 84 | 2MP |
| 4.049 | styrene | 104, 78, 63 | nHex 2MP 3MP | 10.494 | $C_{12}H_{24}$ alkene | 168, 129, 97, 85 | 3MP |
| 4.083 | 1-nonene | 126, 97, 83, 70 | nHex | 10.566 | $C_{12}H_{24}$ alkene | 168, 111, 98, 84 | nHex |
| 4.593 | $C_{10}H_{20}$ alkene | 140, 111, 85, 70 | 3MP | 10.575 | $C_{12}H_{24}$ alkene | 168, 128, 97, 84 | 3MP |
| 4.602 | $C_{10}H_{20}$ alkene | 140, 111, 83, 70 | 3MP | 10.609 | $C_{10}H_{10}$ aromatic | 130, 115 | 2MP 3MP |
| 4.631 | $C_{10}H_{20}$ alkene | 140, 97, 84, 69 | 2MP | 10.878 | $C_{12}H_{24}$ alkene | 168, 97, 83, 70 | 3MP |
| 4.734 | $C_{10}H_{20}$ alkene | 140, 112, 98, 84 | nHex | 10.93 | $C_{11}H_{22}$ alkene | 168, 111, 83, 69 | nHex |
| 4.734 | $C_{10}H_{22}$ branched alkane | 142, 113, 98, 85 | 2MP | 11.033 | $C_{11}H_{22}$ alkene | 168, 141, 113, 85 | 2MP |
| 4.952 | $C_{10}H_{20}$ alkene | 140, 112, 97, 84 | nHex | 11.341 | naphthalene | 128, 102, 75 | nHex 2MP 3MP |
| 4.974 | $C_{10}H_{22}$ branched alkane | 127, 113, 85, 71 | 2MP | 11.803 | $C_{11}H_{22}$ alkene | 168, 140, 111, 97 | nHex |

## SII.4 Selected mass spectra from GC-MS measurements

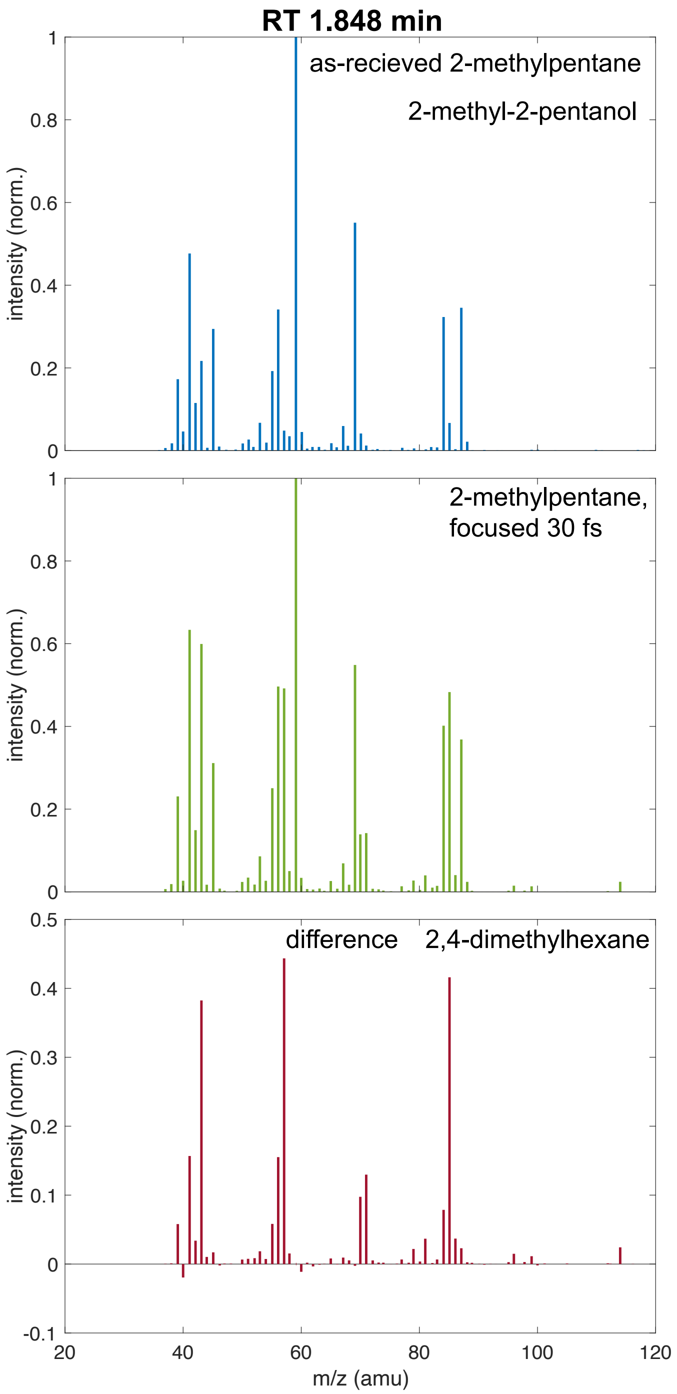


Figure S8: Mass spectra at retention time 1.848 minutes from as-received 2-methylpentane (top) and ablated 2-methylpentane (middle). Subtraction of the as-received 2-methylpentane spectrum resulted in the difference spectrum (bottom). Assignment to 2,4-dimethylhexane made by match to the NIST library.

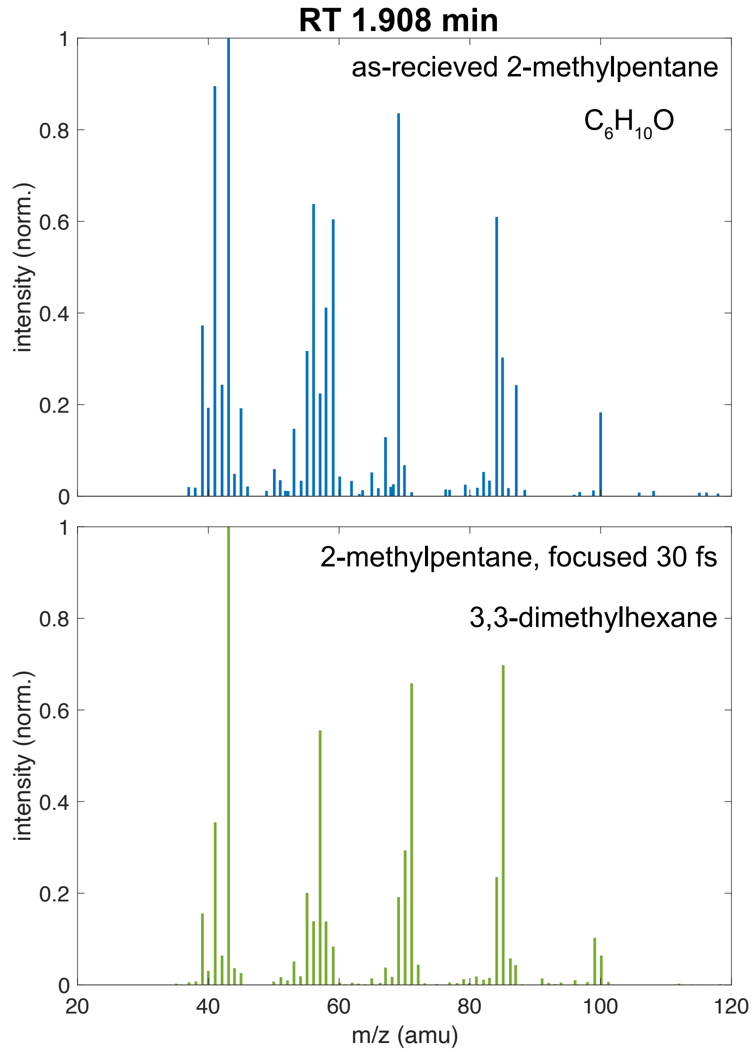


Figure S9: Mass spectra at retention time 1.908 minutes from as-received 2-methylpentane and ablated 2-methylpentane sample. Assignment of 3,3-dimethylhexane in ablated sample made by match to the NIST library.

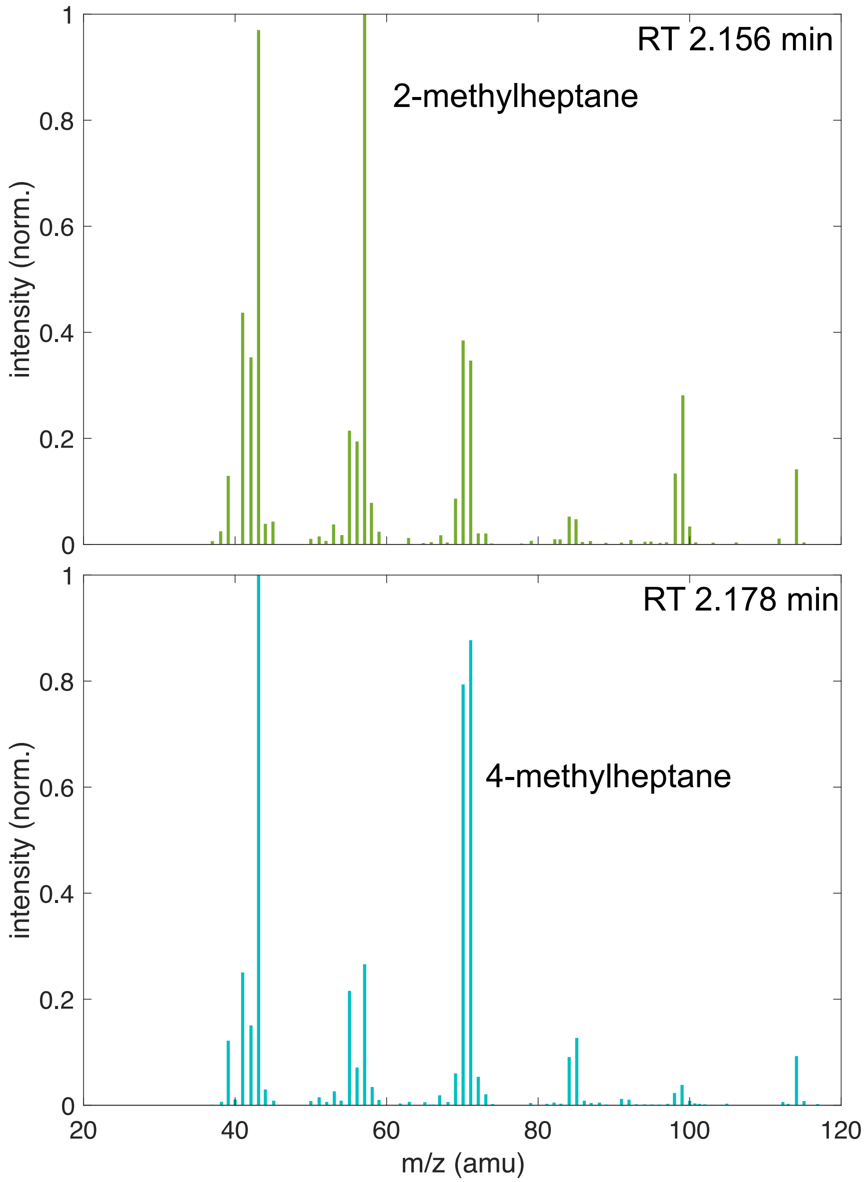


Figure S10: Mass spectra within peak at retention time 2.17 minutes from ablated 2-methylpentane. Assignments made from match to the isoparaffin standard mixture and the NIST library.

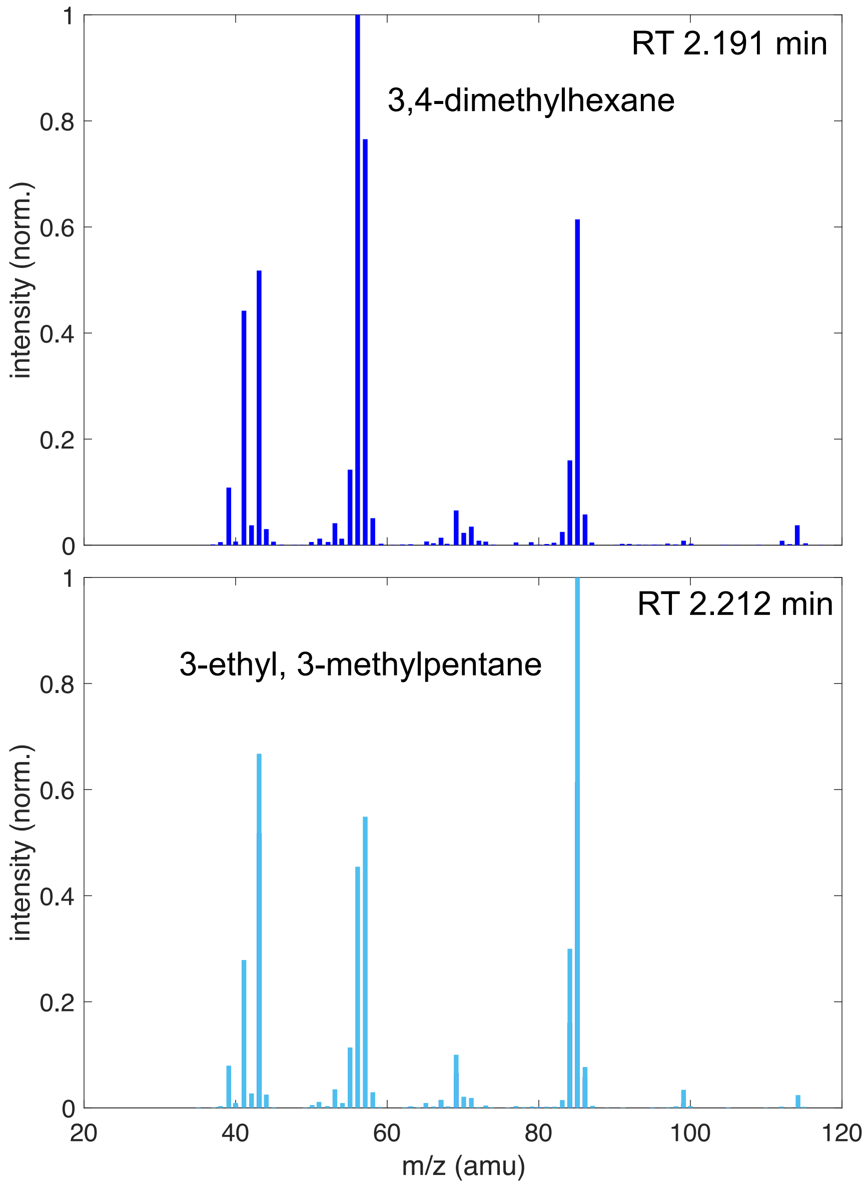


Figure S11: Mass spectra within peak at retention time 2.20 minutes from ablated 3-methylpentane. Assignments made by match to the NIST library.

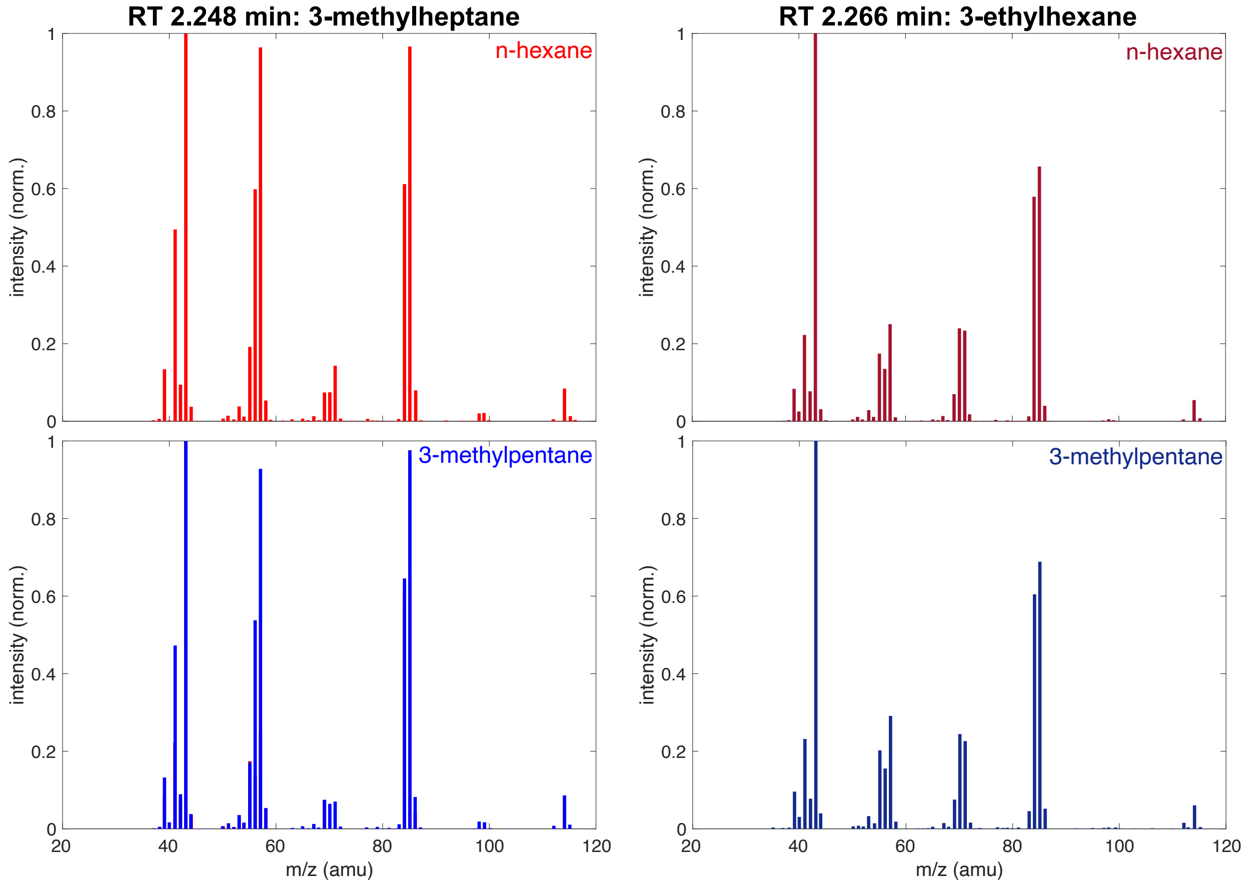


Figure S12: Mass spectra within peak at retention time 2.25 minutes from ablated $n$-hexane and 3-methylpentane. Assignments made from match to the isoparaffin standard mixture and the NIST library.

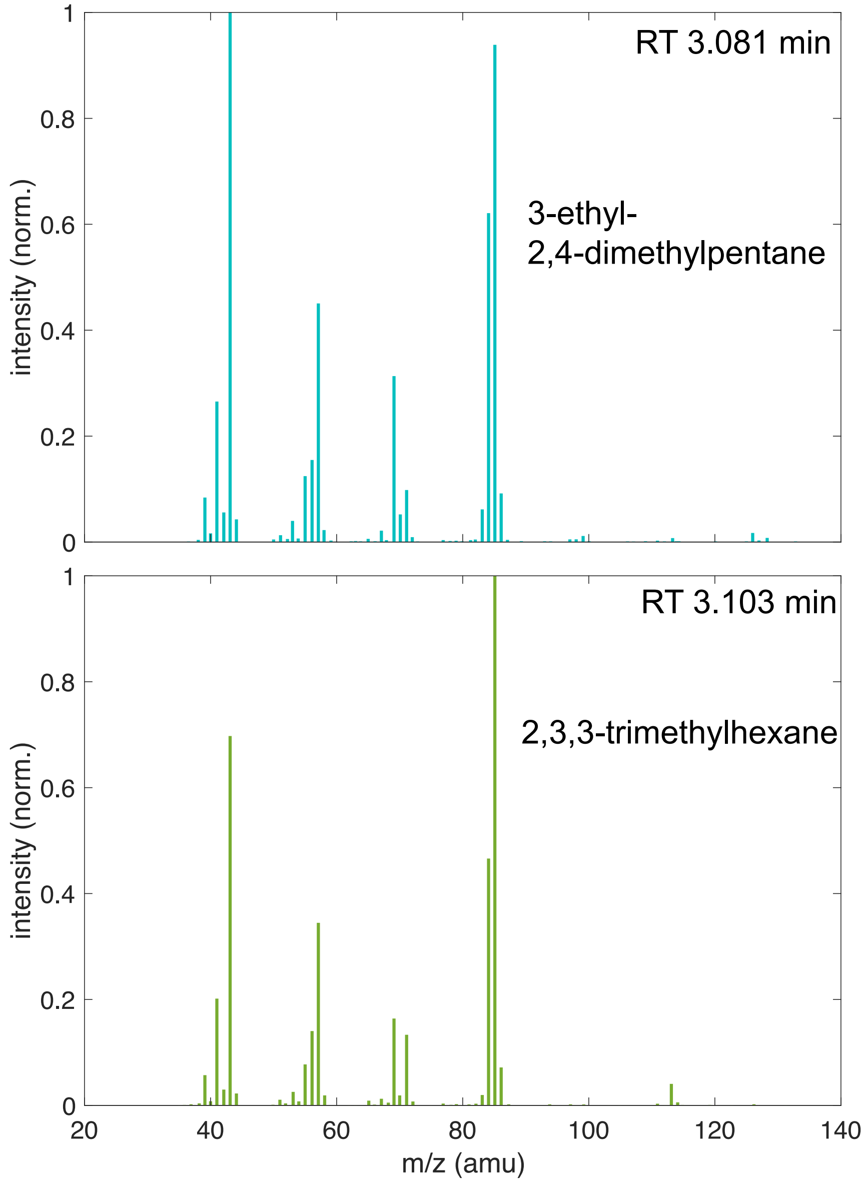


Figure S13: Mass spectra within peak at retention time 3.09 minutes from ablated 2-methylpentane. Assignments made by match to the NIST library.

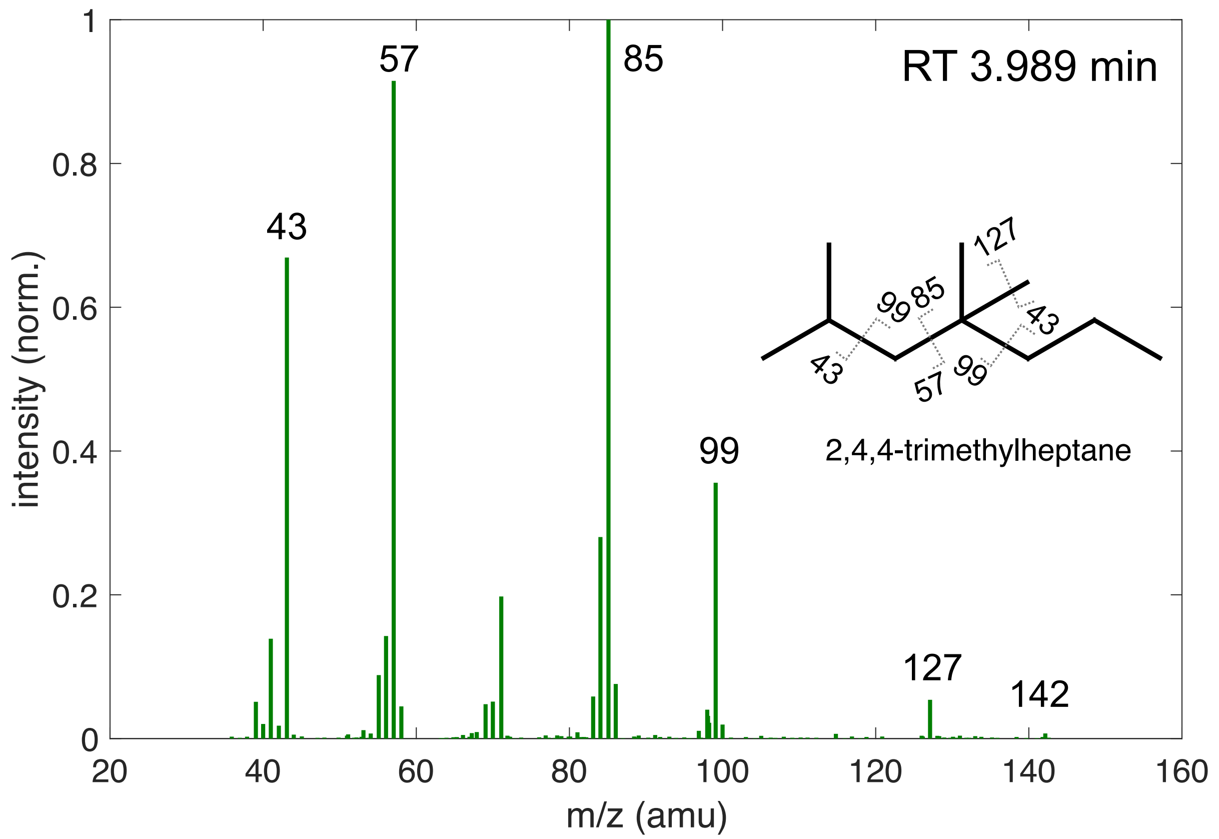


Figure S14: Mass spectrum for the peak at retention time 3.989 minutes from ablated 2-methylpentane. Assignment to 2,4,4-trimethylheptane is based on (1) analysis of $C_{10}H_{22}$ structures expected from addition of an *iso*-butyl radical to a 2-methylpentane molecule (Figure 7 in main text); (2) literature indicating that this isomer elutes after 3-methyloctane (RT 3.728 min, Table S5) and before $n$-nonane (RT 4.255 min, Table S6) in capillary GC-MS;[4] and (3) its Kovats' retention index (RI) of 889. This peak at RT 3.989 min falls between the peaks for 2,4,6-trimethylheptane at RT 3.737 min (Kovats' RI 870) and 3-ethyl-2,5-dimethylhexane at RT 4.203 min (Kovats' RI 891). All Kovats' RI values taken from the NIST chemistry webbook spectral database.[1] Bond cleavages yielding the observed fragment peaks are indicated in the structure.

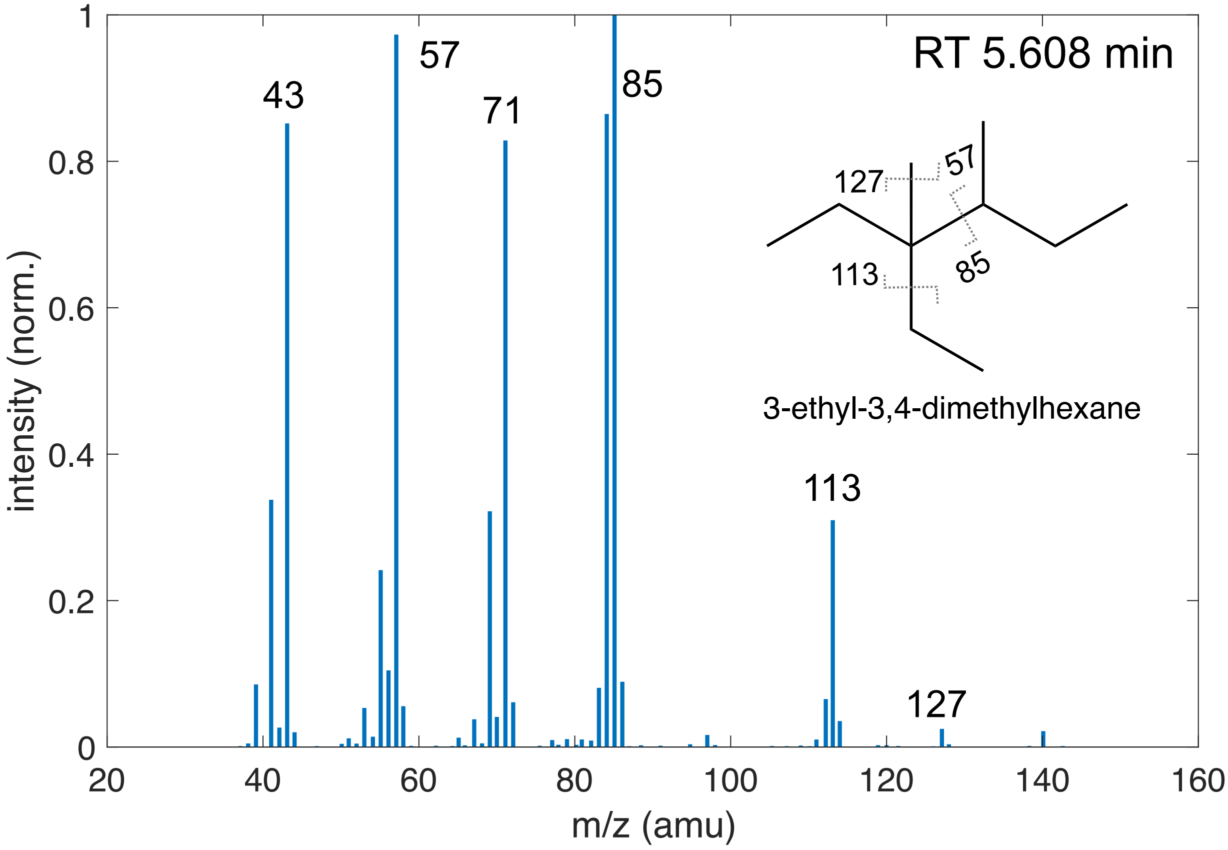


Figure S15: Mass spectrum for the peak at retention time 5.608 minutes from ablated 3-methylpentane. Assignment to 3-ethyl-3,4-dimethylhexane is based on (1) analysis of $C_{10}H_{22}$ structures from addition of a *sec*-butyl radical to 3-methylpentane (Figure 7 in main text) and (2) its Kovats' retention index (RI) of 965. This peak at RT 5.608 min falls to the right of the peaks for 3,4,5-trimethylheptane at RT 5.273 min (Kovats' RI 945) and 5-methylnonane at RT 5.595 min (Kovats' RI 957) produced from $n$-hexane. All Kovats' RI values taken from the NIST chemistry webbook spectral database.[1] Bond cleavages yielding the observed fragment peaks are indicated in the structure. The $m/z$ 71 and 43 peaks are assumed to arise from sequential fragmentation.

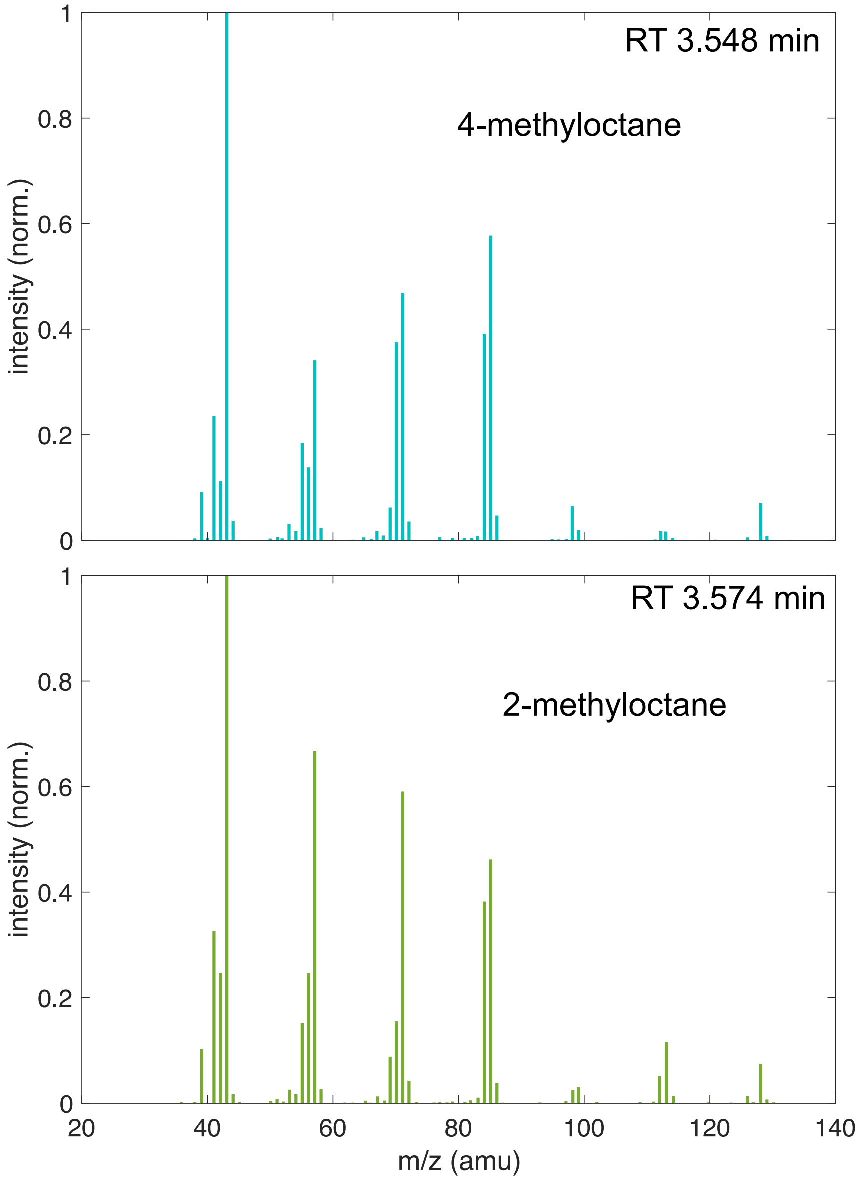


Figure S16: Mass spectra within peak at retention time 3.55 minutes from ablated 2-methylpentane. Assignments to 4-methyloctane and 2-methyloctane made from match to the isoparaffin standard mixture and the NIST library.

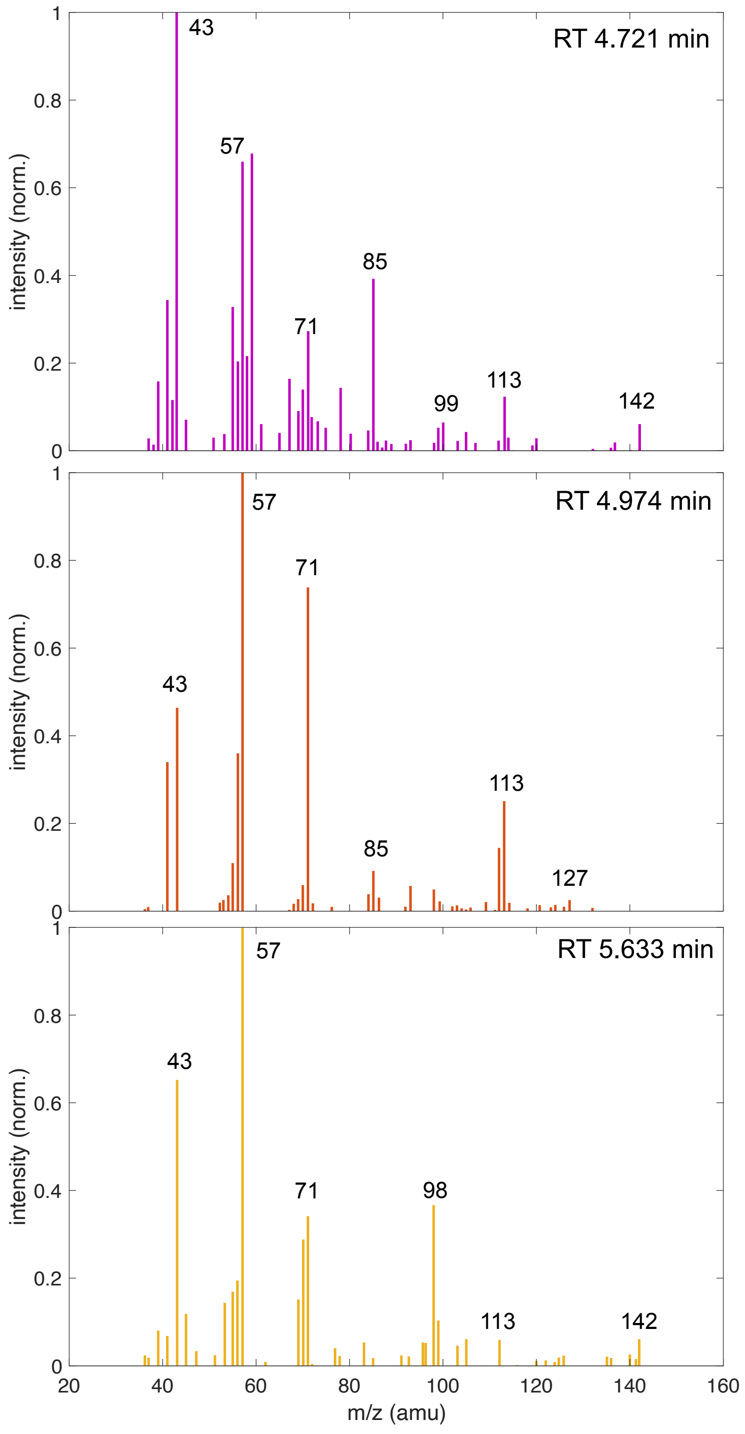


Figure S17: Mass spectra for minor product peaks at RT 4.721, 4.974, and 5.633 min ablated 2-methylpentane. Assignments to undetermined branched $C_{10}H_{22}$ alkanes made based on the fragmentation patterns. These are suspected products arising from reaction between a rearranged *tert*-butyl radical and a 2-methylpentane molecule (Table S10 and Figure S20).

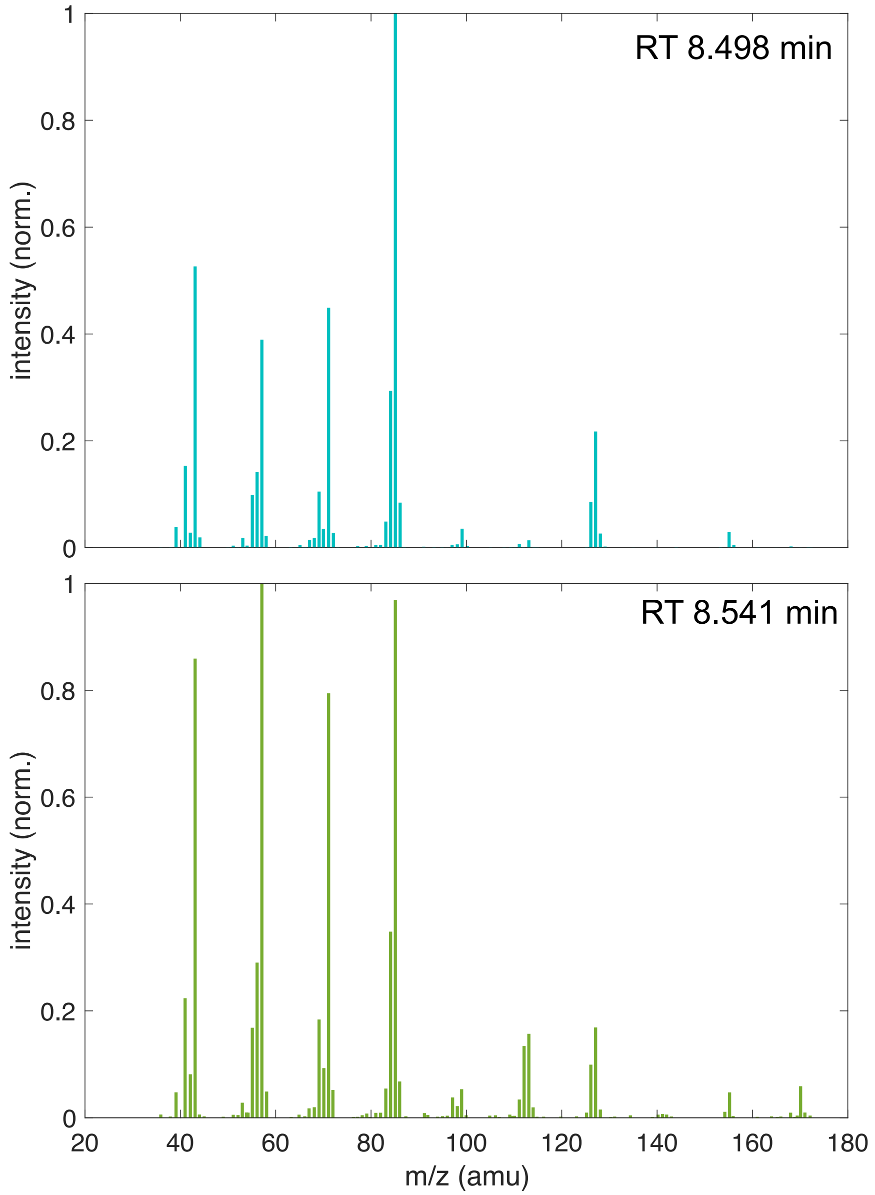


Figure S18: Mass spectra within peak at retention time 8.51 minutes from ablated 2-methylpentane. The $m/z$ 170 peak indicates $C_{12}H_{26}$, but no structural assignments could be made from the NIST library.

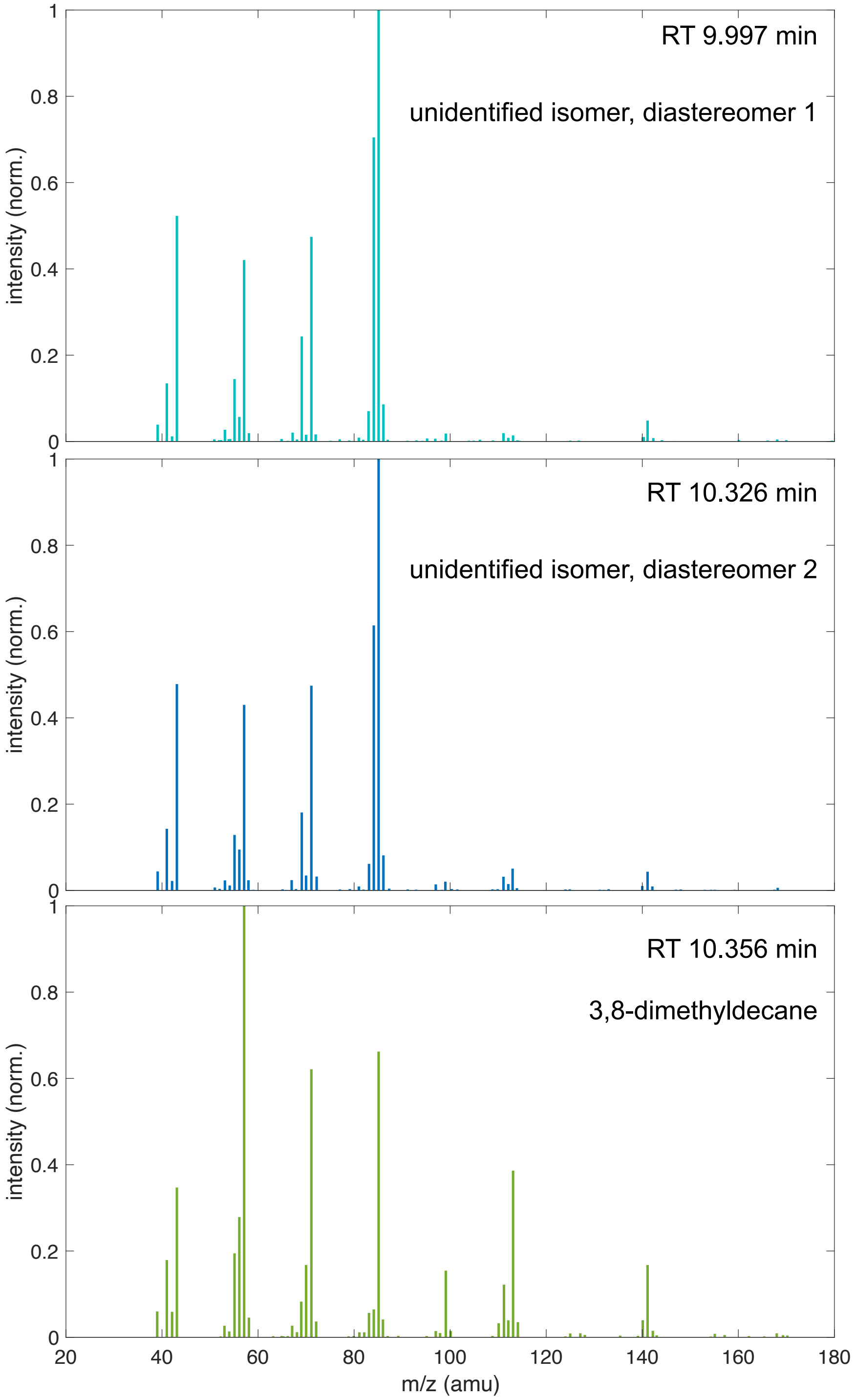


Figure S19: Mass spectra at 9.997 min and on left/right sides of the peak at retention time 10.35 minutes from ablated 3-methylpentane. The peak at 10.356 minutes is assigned to 3,8-dimethyldecane by match to the NIST library. The peaks at 9.997 and 10.322 minutes appear to be diastereomers of a single unidentified $C_{12}H_{26}$ isomer based on the similarity of the mass spectra.

## SII.5 Product distributions from GC-MS

The integrated peak areas of each major and minor product from Tables S6 and S7 were computed for each hexane isomer under each ablation condition (LDP, focused 30 fs, focused 4 ps). All experiments were performed in duplicate. The fractional yields of alkane, alkene, and aromatic products are given in Table S8. The relative yields of each type of alkane product ($C_8H_{18}$, $C_9H_{20}$, $C_{10}H_{22}$, $C_{11}H_{24}$, $C_{12}H_{26}$) are given in Table S9. The relative yields of each observed $C_8H_{18}$, $C_9H_{20}$, and $C_{10}H_{22}$ isomer product, with indications of total products from direct radicals (i.e., that do not undergo rearrangement) and rearranged radicals, are given in Table S10.

Table S8: Relative yields (%) of product classes detected in GC-MS. Error bars denote standard deviation from two separate experiments at each condition.

| Product | LDP | 30 fs | 4 ps |
|---|---|---|---|
| | | *n*-hexane | |
| alkanes | 97.7±0.9 | 97.2±1.8 | 92.5±0.9 |
| alkenes | 2.1±0.6 | 2.6±1.3 | 6.0±0.8 |
| aromatics | 0.2±0.1 | 0.2±0.1 | 1.5±0.1 |
| | | 2-methylpentane | |
| alkanes | 98.1±0.7 | 97.5±0.2 | 92.3±0.9 |
| alkenes | 1.6±0.1 | 2.4±0.2 | 5.7±0.9 |
| aromatics | 0.3±0.2 | 0.1±0.1 | 2.0±0.1 |
| | | 3-methylpentane | |
| alkanes | 97.3±0.6 | 95.2±0.9 | 91.0±0.6 |
| alkenes | 2.6±0.4 | 4.7±0.7 | 7.7±0.5 |
| aromatics | 0.1±0.1 | 0.1±0.1 | 1.3±0.1 |

Table S9: Relative yields (%) of $C_8H_{18}$–$C_{12}H_{26}$ alkanes detected in GC-MS. Error bars denote standard deviation from two separate experiments at each condition.

| Product | LDP | 30 fs | 4 ps |
|---|---|---|---|
| | *n*-hexane | | |
| $C_8H_{18}$ | 10.3±0.4 | 10.6±0.4 | 11.7±0.8 |
| $C_9H_{20}$ | 11.1±0.2 | 11.3±0.2 | 10.3±0.1 |
| $C_{10}H_{22}$ | 12.4±0.1 | 12.2±0.2 | 8.5±0.2 |
| $C_{11}H_{24}$ | 1.4±0.1 | 1.5±0.2 | 1.2±0.1 |
| $C_{12}H_{26}$ | 64.6±0.4 | 64.4±0.2 | 68.4±0.6 |
| | 2-methylpentane | | |
| $C_8H_{18}$ | 7.0±0.5 | 5.7±1.3 | 6.9±0.1 |
| $C_9H_{20}$ | 35.4±0.1 | 34.1±0.1 | 24.7±0.8 |
| $C_{10}H_{22}$ | 3.1±0.3 | 3.2±0.2 | 3.5±0.2 |
| $C_{11}H_{24}$ | 3.8±0.2 | 4.3±0.3 | 4.7±0.3 |
| $C_{12}H_{26}$ | 50.6±0.4 | 52.5±0.8 | 60.2±1.4 |
| | 3-methylpentane | | |
| $C_8H_{18}$ | 26.8±2.8 | 23.0±1.2 | 23.2±0.2 |
| $C_9H_{20}$ | 0.4±0.1 | 0.6±0.1 | 0.8±0.1 |
| $C_{10}H_{22}$ | 22.9±1.1 | 21.6±0.7 | 15.9±0.3 |
| $C_{11}H_{24}$ | 1.8±0.1 | 2.4±0.3 | 2.7±0.1 |
| $C_{12}H_{26}$ | 48.1±3.8 | 52.4±1.5 | 57.3±0.6 |

Table S10: Relative yields (%) of $C_8H_{18}$, $C_9H_{20}$, and $C_{10}H_{22}$ isomers detected in GC-MS. Error bars denote standard deviation from two separate experiments at each condition. Isomers listed in red text are produced by reactions of rearranged radicals with the parent hexane molecule (see Figure S20).

**n-hexane**

| product | LDP | 30 fs | 4 ps |
|---|---|---|---|
| 3-methylheptane<br>3-ethylhexane | 21.0±1.0 | 21.0±1.2 | 26.6±2.1 |
| n-octane | 9.5±0.3 | 10.0±0.1 | 11.9±0.5 |
| 3-ethyl-2-methylhexane | 0.4±0.1 | 0.6±0.2 | 1.7±0.1 |
| 2,3-dimethylheptane | 0.3±0.1 | 0.4±0.2 | 1.2±0.2 |
| 4-ethylheptane | 11.4±0.2 | 11.2±0.3 | 10.3±0.2 |
| 4-methyloctane | 12.3±0.3 | 12.1±0.4 | 11.8±0.2 |
| n-nonane | 8.5±0.2 | 8.7±0.1 | 8.1±0.1 |
| 3,4-dimethyloctane | 0.3±0.1 | 0.3±0.1 | 1.0±0.1 |
| 4-ethyloctane | 13.3±0.1 | 13.0±0.1 | 9.1±0.3 |
| 5-methylnonane | 13.6±0.2 | 13.1±0.1 | 9.3±0.1 |
| 3-methylnonane | 0.4±0.1 | 0.5±0.1 | 1.6±0.1 |
| n-decane | 9.0±0.2 | 9.1±0.5 | 6.6±0.2 |
| **total no rearrangement** | **98.6±0.4** | **98.3±0.6** | **94.3±0.3** |
| **total rearrangement** | **1.4±0.4** | **1.7±0.6** | **5.7±0.3** |

**2-methylpentane**

| product | LDP | 30 fs | 4 ps |
|---|---|---|---|
| 3,3-dimethylhexane | 5.7±0.9 | 5.3±0.1 | 3.9±0.5 |
| 3-ethyl,2-methylpentane | 4.7±0.4 | 2.8±2.5 | 4.9±0.4 |
| 2-methylheptane<br>4-methylheptane | 5.1±0.3 | 5.3±0.6 | 10.8±0.6 |
| 2,3,5-trimethylhexane | 6.5±0.1 | 6.7±0.2 | 8.9±0.7 |
| 2,4-dimethylheptane | 18.0±0.6 | 17.9±0.1 | 16.6±2.1 |
| 4,4-dimethylheptane | 9.2±0.2 | 9.2±0.1 | 4.9±0.1 |
| 2,6-dimethylheptane | 4.1±0.1 | 4.6±0.1 | 5.5±0.4 |
| 3-ethyl-2,4-dimethylpentane<br>2,3,3-trimethylhexane | 12.9±0.2 | 12.9±0.2 | 10.6±0.6 |
| 3-ethyl-2-methylhexane | 9.0±0.1 | 9.1±0.2 | 7.0±0.2 |
| 4-methyloctane<br>2-methyloctane | 18.2±0.2 | 18.8±0.1 | 16.9±1.2 |
| 2,4,6-trimethylheptane | 1.0±0.1 | 1.3±0.1 | 1.4±0.1 |
| 2,4,4-trimethylheptane | 1.1±0.1 | 1.2±0.1 | 1.5±0.1 |
| 3-ethyl-2,5-dimethylhexane | 1.1±0.1 | 1.3±0.1 | 1.5±0.1 |
| unidentified $C_{10}H_{22}$ | 0.3±0.2 | 0.1±0.1 | 0.6±0.1 |
| 2,5-dimethyloctane | 2.1±0.2 | 2.4±0.2 | 2.8±0.1 |
| 2,7-dimethyloctane | 1.0±0.1 | 1.1±0.1 | 1.8±0.1 |
| unidentified $C_{10}H_{22}$ | 0 | 0.1±0.1 | 0.3±0.1 |
| unidentified $C_{10}H_{22}$ | 0.1±0.1 | 0 | 0.2±0.1 |
| **total no rearrangement** | **99.6±0.4** | **99.8±0.2** | **98.9±0.2** |
| **total rearrangement** | **0.4±0.4** | **0.2±0.2** | **1.1±0.2** |

**3-methylpentane**

| product | LDP | 30 fs | 4 ps |
|---|---|---|---|
| 3,4-dimethylhexane<br>3-ethyl,3-methylpentane | 32.4±3.9 | 29.3±2.3 | 30.6±0.3 |
| 3-methylheptane<br>3-ethylhexane | 21.1±1.7 | 21.6±0.4 | 27.5±0.2 |
| 2,5-dimethylheptane | 0.5±0.1 | 0.7±0.1 | 0.9±0.2 |
| 3,4-dimethylheptane | 0.2±0.1 | 0.3±0.1 | 0.6±0.1 |
| 3-methyloctane | 0.1±0.1 | 0.3±0.1 | 0.7±0.1 |
| 3-ethyl-5-methylheptane | 6.9±0.2 | 7.4±0.1 | 6.2±0.1 |
| 3,6-dimethyloctane | 13.0±0.7 | 14.1±0.1 | 13.2±0.2 |
| 3,4,5-trimethylheptane | 16.8±1.0 | 17.3±0.6 | 14.3±0.4 |
| 2,4,4-trimethylheptane | 9.0±0.3 | 9.0±0.8 | 6.1±0.1 |
| **total no rearrangement** | **99.2±0.2** | **98.7±0.3** | **97.9±0.2** |
| **total rearrangement** | **0.8±0.2** | **1.3±0.3** | **2.1±0.2** |

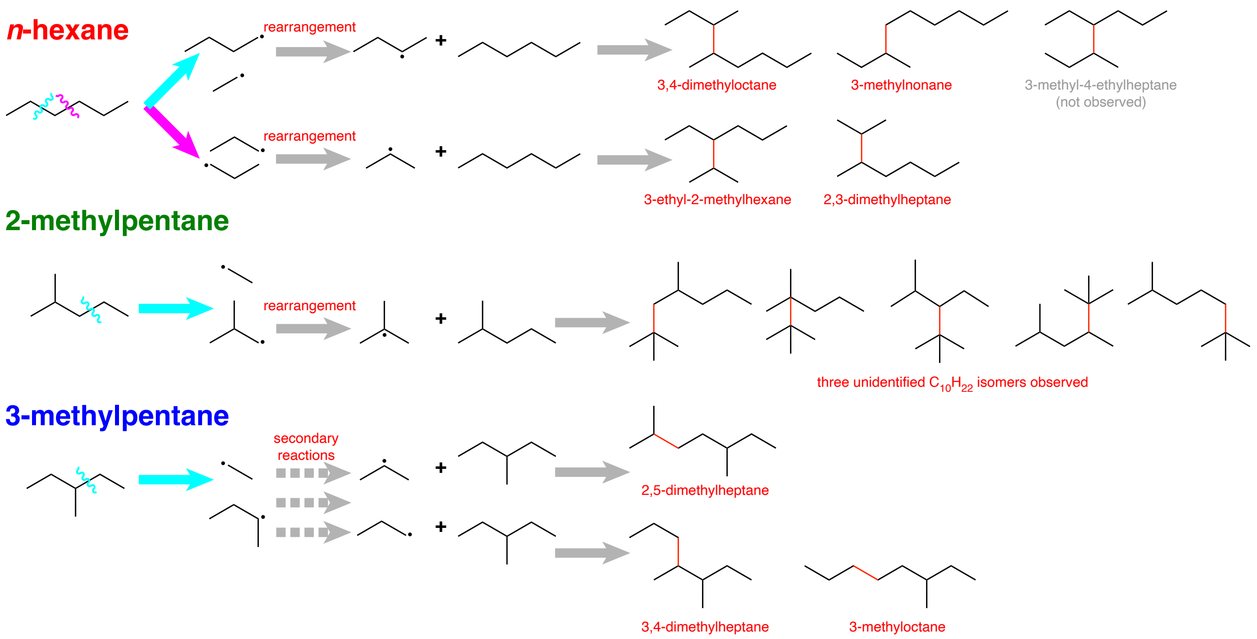


Figure S20: Potential reaction pathways leading to the products arising from rearranged radicals or secondary reactions. In *n*-hexane, the initially produced *n*-propyl radical would have to rearrange to the more stable *iso*-propyl radical to produce 3-ethyl-2-methylhexane and 2,3-dimethylheptane, while the *n*-butyl radical would rearrange to the more stable *sec*-butyl radical to produce 3,4-dimethyloctane and 3-methylnonane. Reaction between the *sec*-butyl radical and a *n*-hexane molecule could also produce 3-methyl-4-ethylheptane, but this structure was not observed in GC-MS. In 2-methylpentane, the initially formed *iso*-butyl radical could rearrange to the highly stable *tert*-butyl radical to produce the unidentified $C_{10}H_{22}$ isomers observed in GC-MS; the 5 possible structures are shown. In 3-methylpentane, the formation of $C_9H_{20}$ structures requires secondary reactions of the initially formed *sec*-butyl and ethyl radicals to produce *n*-propyl and *iso*-propyl radicals. Reaction between 3-methylpentane and the *iso*-propyl radical produces the observed 2,5-dimethylheptane, while reaction with the *n*-propyl radical produces the observed 3,4-dimethylheptane and 3-methyloctane. Other possible product structures that were not observed in GC-MS are not shown.

# References


(1) NIST Standard Reference Database 69. http://webbook.nist.gov/chemistry/, Last checked 07/01/2024.

(2) Frias Batista, L. M.; Moody, M.; Weththasingha, C.; Kaplan, E.; Faruque, I.; El-Shall, M. S.; Tibbetts, K. M. Understanding photochemical pathways of laser-induced metal ion reduction through byproduct analysis. *Phys. Chem. Chem. Phys.* **2023**, *25*, 18844–18853.

(3) Frias Batista, L. M.; Kaplan, E.; Weththasingha, C.; Cook, B.; Harris, S.; Nag, A.; Tibbetts, K. M. How Pulse Width Affects Laser Ablation of Organic Liquids. *The Journal of Physical Chemistry B* **2023**, *127*, 6551–6561.

(4) Sanders, W. N.; Maynard, J. B. Capillary gas chromatographic method for determining the C3-C12 hydrocarbons in full-range motor gasolines. *Analytical Chemistry* **1968**, *40*, 527–535.
